# A comprehensive analysis of female participation in cardiovascular trials involving the WCN investigator network

**DOI:** 10.1007/s12471-025-01999-4

**Published:** 2025-11-12

**Authors:** Marte F. van der Bijl, Jeanine E. Roeters van Lennep, Astrid Schut, Iris C. D. Westendorp, Yolande Appelman, Hester M. den Ruijter, Eric Boersma

**Affiliations:** 1 Department of Cardiology, Cardiovascular Institute, Erasmus MC, Rotterdam, The Netherlands; 2 Department of Internal Medicine, Cardiovascular Institute, Erasmus MC, Rotterdam, The Netherlands; 3 Dutch Network for Cardiovascular Research, Utrecht, The Netherlands; 4 Department of Cardiology, Red Cross Hospital, Beverwijk, The Netherlands; 5 Department of Cardiology, Amsterdam Heart Centre, Amsterdam Cardiovascular Sciences, VU University Medical Center, Amsterdam, The Netherlands; 6 Laboratory for Experimental Cardiology, Department of Cardiology, University Medical Center Utrecht, Utrecht, The Netherlands

**Keywords:** Cardiovascular Diseases, Clinical Trials as Topic, Sex, Women’s Health

## Abstract

**Background:**

Prior studies showed underrepresentation of females in cardiovascular disease (CVD) clinical trials, potentially hindering accurate treatment effect estimates. We assessed the female contribution to treatment effect estimates in selected CVD trials and explored sex differences in efficacy outcomes.

**Methods:**

We analyzed completed (1997–2024) randomized controlled CVD trials performed via the Dutch WCN Investigator Network. Female participation was quantified using the Participation to Prevalence (in the population) Ratio (PPR_F_). In trials with a cardiovascular event as the primary efficacy endpoint, a meta-analysis was conducted to evaluate differences in treatment effect on the study-specific primary endpoint between females and males using a random-effects model.

**Results:**

In 115 trials investigating various treatments across different cardiovascular domains (801 k participants, 29.1% females), the median PPR_F_ was 0.75 (interquartile range: 0.64–0.83), while 58% of trials had a PPR_F_ below 0.8 (underrepresentation). Based on 46 trials, female contribution to primary endpoints was lower than their sample size contribution (mean 26.2% versus 28.5%). Similarly, based on 66 trials, female contribution to sex-stratified efficacy estimates was lower than their sample size contribution (27.4% versus 29.2%). Regarding the primary endpoint, the relative treatment effect was similar in females and males: pooled difference of the relative effect measure on the natural log scale of −0.02, 95% CI −0.05 to 0.01, *p* = 0.23, I^2^ = 11%.

**Conclusion:**

Despite underrepresentation, female participation in the selected WCN-CVD trials was sufficient to exclude major sex differences in efficacy. Given the limited and heterogeneous trial sample, further disease-specific studies are needed, and greater female inclusion remains essential for equity and safety insights.

**Supplementary Information:**

The online version of this article (10.1007/s12471-025-01999-4) contains supplementary material, which is available to authorized users.

## What’s new?

- Females contributed, on average, 27.4% to the estimated treatment effect, which was lower than their contribution to the total sample size (29.1%).
- The above-mentioned discrepancy is primarily due to the lower incidence of primary endpoints in females compared to males.
- In response to growing and welcome emphasis on sex-stratified reporting, this study meta-analyzes pooled treatment effects and finds no evidence of systematic differences favoring either females or males.

## Introduction

Although cardiovascular disease (CVD) is associated with high prevalence, morbidity, and mortality [1–4], females remain significantly underrepresented in cardiovascular clinical trials relative to their disease burden [5, 6]. This underrepresentation varies by disease type. For example, the participation to prevalence (in the population) ratio for females (PPR_F_) is lower in trials for heart failure and coronary artery disease trials compared with hypertension [5, 6]. Such underrepresentation may hinder accurate assessment of treatment efficacy and safety for females and limit the generalizability of trial results [7, 8]. This issue is particularly relevant in sex-specific pathophysiology, such as higher ejection fraction in women with heart failure [9] and sex-related differences in coronary artery disease [10, 11].

In the current manuscript, we aim to obtain insight into the contribution of females to estimates of treatment effect in the cardiovascular domain. We further study the efficacy estimates between females and males that were observed in CVD randomized clinical trials (RCTs) performed via the WCN investigator network. Our analysis goes beyond the assessment of female representation in CVD trials based on the PPR [5, 6]. Although relevant, we do not address safety, side effects, or ethical issues [12].

## Methods

### Terminology

In the context of this study, sex is defined as biological sex as reported by the trials and is hence associated with physical and physiological attributes [13]. Therefore, we will use the terms female and male throughout the manuscript [13]. Due to the way sex was reported in the trials, our analysis is limited to a binary classification (female vs male) and does not account for other sexes or gender-related aspects.

### Clinical trial selection

We assessed all completed RCTs performed via the Dutch WCN Investigator Network from the first publication of a completed study in 1997 through November 4, 2024, as listed on their website [14]. All trials received approval from an Institutional Review Board (IRB) and/or a medical ethics committee, as appropriate. These trials encompassed both pharmacological and non-pharmacological interventions across a broad spectrum of CVD populations. We included only RCTs that were registered in a clinical trial registry and had published results in English peer-reviewed journals. We excluded long-term (open-label) extension studies.

### Data collection

Based on the main publications, for each trial, we obtained the following information: target patient population, experimental treatment, control treatment, primary efficacy endpoint, and the number of females and males randomized. For trials with a primary efficacy endpoint that was defined as a (composite of) clinical event(s) we also obtained the number of females and males who reached the endpoint, as well as the sex-specific relative efficacy measure (REM) (i.e. hazard ratio [HR], relative risk [RR] or odds ratio [OR]) with 95% confidence interval (CI). If sex-specific REMs were not presented, we determined ORs based on the available absolute numbers of females and males with/without the primary efficacy endpoint.

Across all trials, four target populations emerged: patients with arrhythmias, atherosclerotic cardiovascular disease (ASCVD), diabetes mellitus, or heart failure. The PPR_F_ for acute myocardial infarction, angina pectoris, atrial fibrillation, general CVD, general heart failure, and pacemaker/implantable cardioverter defibrillators use was obtained from the Netherlands Heart Registration (NHR) [15]. For acute coronary syndrome, coronary heart disease, and diabetes mellitus, prevalence ratios were derived from Statistics Netherlands (Dutch: Centraal Bureau voor de Statistiek; CBS) [16]. In the absence of Dutch prevalence data, international data from Swedish and American studies were used for heart failure subphenotypes (HFrEF and HFpEF) and supraventricular tachycardia. An overview of the prevalence data utilized can be found in the Electronic Supplementary Material (ESM) Table S1. Trials with a factorial design, in which multiple treatments were compared, had all treatment arms extracted and included in the analysis.

## Statistical data analysis

For each study, we determined the PPR_F_, calculated as the female/male inclusion ratio divided by the female/male prevalence ratio of the corresponding disease in the population. We describe these PPRs, in line with existing literature, which considers a PPR between 0.8 and 1.2 an adequate representation, whereas PPRs below or above this range indicate under- or overrepresentation, respectively [5, 6].

The amount of information contained in a statistic is determined by its standard error. Therefore, the inverse of the squared standard error (variance) is often used as a weighting factor when deriving stratified estimates within a study or pooling statistics across multiple studies [17]. Consequently, in stratified or pooled estimates, statistics with low variance receive a relatively high weight. Moreover, without further explanation, stratified or pooled estimates of ratio measures for clinical endpoints are calculated using the logarithm of that measure and its standard error [18]. Notably, these standard errors are influenced not only by the sample size, but also by the number of patients who reach the endpoint.

Against this theoretical background, for the trials with a (composite of) clinical event(s) as the primary efficacy endpoint, we calculated the sex-specific natural logarithm of the REM (logREM) of the experimental treatment along with their inverse variances, which, as explained, indicate their ‘statistical weight’. Subsequently, based on these weights, for each trial, we quantified the contribution of females to a sex-stratified estimate of the REM. Furthermore, we determined the difference in logREM between males and females (delta logREM), which we then meta-analyzed to obtain a pooled estimate using a random-effects model. Note that a delta logREM = 0 implies no difference in efficacy of the experimental treatment (relative to control treatment) between males and females. We therefore tested the null hypothesis H0: pooled delta logREM = 0 against the alternative hypothesis H1: pooled delta logREM ≠ 0. A *p*-value < 0.05 was considered statistically significant. To assess publication bias, we used a funnel plot and Egger’s test [19].

The analyses and visualizations of the results were conducted using the ggplot2 and meta packages in R, version 4.3.1.

## Results

**Fig. 1:**
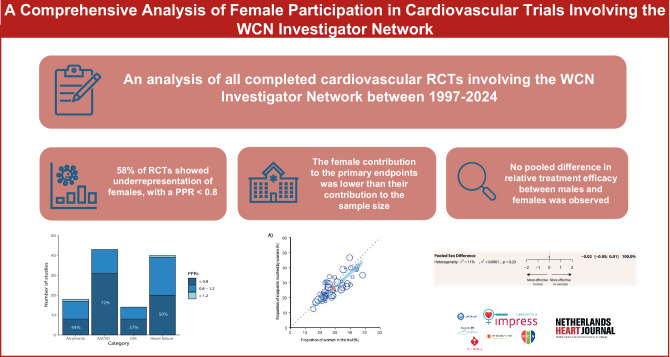
Infographic

In the period 1997–2024, a total of 165 WCN trials were completed, of which 115 met our inclusion criteria (see ESM Figure S1), totaling over 801,000 participants. The most recent manuscript was published in November 2023 (Fig. 1).

### Participation to prevalence ratio

The percentage of participating females varied from 14.6 to 71.2%, with an average of 29.1%. Most trials were performed in the domain of ASCVD (43 trials), followed by heart failure (40 trials), arrhythmia (18 trials) and diabetes mellitus (14 trials). The median PPR_F_ was 0.75 [1st–3rd quartiles: 0.64–0.83] (see ESM Table S2). The PPR_F_ was < 0.8 (underrepresentation of females) in 58% of all trials, and was highest—72%—in trials within the domain of ASCVD (see ESM Figure S2). A PPR_F_ > 1.2 (overrepresentation of females) was observed in 2% of trials.

### Contribution of females to the estimated relative effect measure

**Fig. 2:**
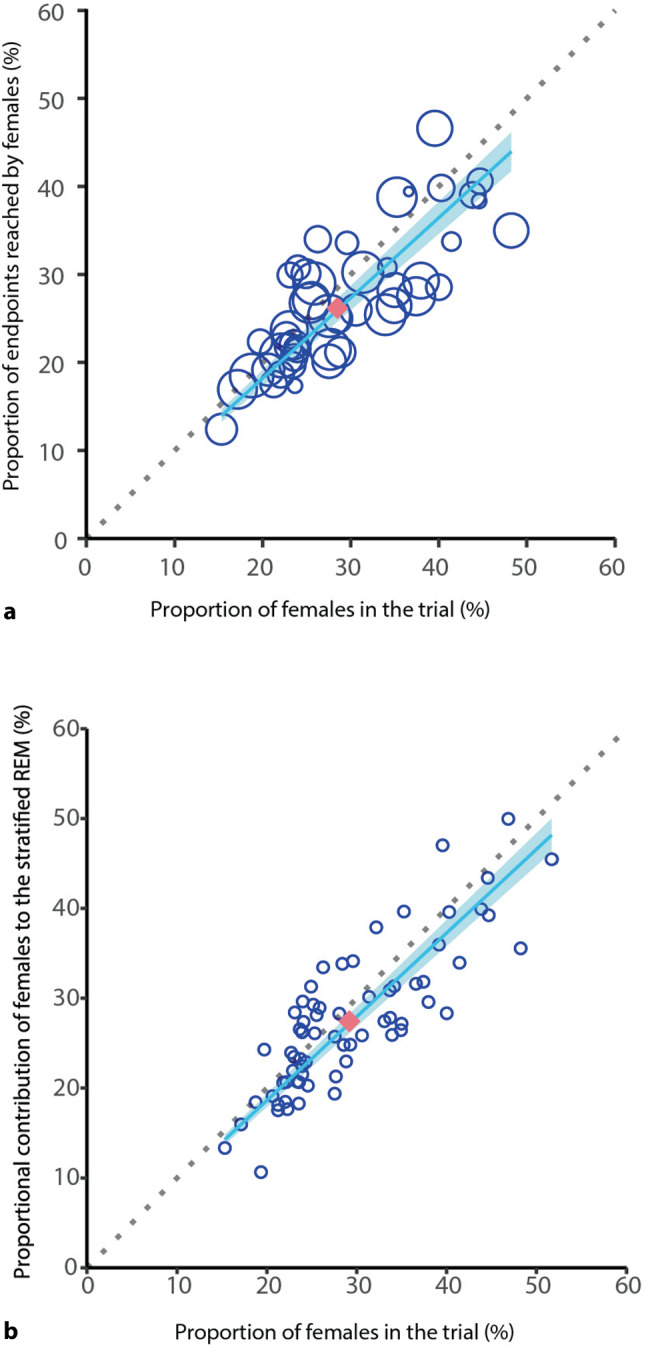
**a** Relationship between proportion of female participants and endpoints reached by females. Dotted line (X,Y); solid line regression line. Size of the dots: study weight (1/variance). **b** Relationship between proportion of female participants and proportional contribution of females to the sex-stratified REM. Dotted line (X,Y); solid line regression line

As demonstrated in Fig. 2a, in 33 out of 46 trials (with a total of 48 comparisons), the percentage of females among those who reached the primary efficacy endpoint was smaller than the percentage of females included in the trial. The mean weighted proportion in endpoints was 26.2% versus the expected 28.5%. This discrepancy increased with larger sample sizes (fixed intercept at 0, beta = 0.91, *p* < 0.0001). Figure 2b illustrates that, in 45 (68.2%) out of 66 trials (with a total of 69 comparisons), the percentage contribution of females to the sex-stratified estimate of the REM of the experimental treatment was also lower than the percentage of females included. The mean proportional contribution of females was 27.4% versus the expected 29.2%. Similarly, this difference was associated with sample size (fixed intercept at 0, beta = 0.93, *p* < 0.0001).

### Differential efficacy in the primary endpoint between females and males

**Fig. 3:**
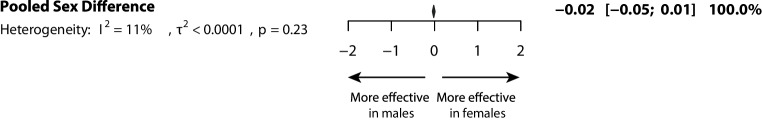
Forest plot pooled difference log(REM)

The sex difference in the effect of the experimental treatment in terms of the primary efficacy endpoint (delta log(REM)) could be obtained in 66 trials (680 k participants; 29% females). With a total of 69 comparisons between experimental treatment and placebo or standard of care, and different efficacy endpoints (see ESM Table S3A). In these trials, which included 85% of participants in all *N* = 115 trials, the pooled REM (in 61 out of 66 trials provided as an HR, see ESM Table S3B) in men was 0.88 (95% CI 0.85–0.90) and in females 0.89 (95% CI 0.85–0.92). The pooled delta log (REM) was −0.02, with 95% CI ranging from −0.05 to 0.01 (*p*-value 0.23; Fig. 3 shows the pooled result, and Figure S3 presents individual trial-level data), indicating no evidence for a differential effect of the experimental treatment on the primary efficacy endpoint between females and males. Heterogeneity was largely absent (I^2^ 11%), and there was no sign of publication bias (*p*-value of the Egger’s test 0.82; see ESM Figure S4).

## Discussion

Our study of trials involving the Dutch WCN Investigator Network confirms previous reports showing that females are underrepresented in randomized cardiovascular intervention trials. In our study, this underrepresentation was most prominent in the domain of ASCVD. In most trials, the contribution of females to the estimated treatment effect was even lower than their contribution to the sample size due to the lower incidence of the primary endpoint. Nevertheless, we found no indication of a differential effect between females and males on the primary endpoint. Importantly, the likelihood of a Type II error appears low, given that the pooled sex difference (delta log(REM)) was close to zero with a narrow confidence interval.

Numerous arguments have been proposed to diversify the study populations of RCTs [12], including the potential to uncover differences in treatment outcomes between females and males. However, RCTs are typically designed to quantify a homogeneous treatment effect across the entire study population. Consequently, subgroup sample sizes are often insufficient to assess treatment efficacy adequately within specific groups [12]. While sex-stratified reporting in individual trials is a positive and necessary development, it is important to interpret these individual trial results with caution. When trials are not specifically powered to detect sex-based differences, due to the absence of a prespecified hypothesis or expectation of systematic variation, any apparent differences may be due to random variation (′chance′). Our pooled analysis provides no evidence of a consistent difference in treatment efficacy between sexes, either overall or within specific disease domains, underscoring the need for careful interpretation of individual trial results in this context and providing reassurance about the current evidence. If the focus is solely on generating evidence for treatment efficacy, these results may suggest that including more females in such trials is not strictly necessary. However, safety and adverse drug reactions (ADRs) are also critical in treatment decisions. Watson et al. reported that females experience more ADRs than males [20], yet sex-specific safety reporting is often lacking in, for example, HF trials [21]. Because phase 3 trials are primarily designed to evaluate efficacy, they may not adequately capture sex-related differences in safety outcomes. Future research should prioritize representative observational data to more effectively assess safety across diverse populations.

Beyond detecting potential heterogeneous effects, there is considerable value in including more females in all phases of cardiovascular trials. Confidence in research findings may require that patients see themselves reflected in the study population [22, 23]. Additionally, diversifying trial populations is also relevant from an ethical point of view, as participation in clinical trials can offer immediate benefits to participants and facilitate the rapid adoption of trial results in currently underrepresented groups, such as females [23]. To achieve this goal, future studies should focus on the reasons why females are less likely to participate in cardiovascular trials.

It is noteworthy that in the majority of studies, a lower proportion of females reached the study endpoint compared to their contribution to the sample size. The lower incidence of (cardiovascular) events among females during the follow-up period prompts inquiry into the baseline characteristics of these females in comparison to men. One possible explanation is that females enrolled in the trials may have had more favorable cardiovascular risk profiles compared to their male counterparts, potentially resulting in fewer events. Alternatively, early dropout from the study, potentially attributable to side effects or personal reasons, may prevent females from reaching the study endpoint. This merits further investigation, particularly through an intersectional lens that considers not only biological sex but also gender and sociocultural factors.

### Strengths and limitations

A key strength of our study is that it explores the underrepresentation of females beyond the PPR. Specifically, across the full spectrum of CVD, we examined the sex-specific contribution to the primary endpoint in relevant clinical trials and quantified sex differences in efficacy measures. This is important, as the PPR has limitations due to its reliance on prevalence data, which may not reflect temporal changes or study-specific eligibility criteria. Another limitation is the variation in clinical endpoints (see ESM Table 3A). Although this does not affect the internal validity of our study, as we focused on differences in relative outcome measures, it may somewhat limit the generalizability of our findings. The primary caveat of our work is the incidental selection of WCN studies assessing diverse treatments across heterogeneous cardiovascular subdomains, each represented by only a small number of trials. In that respect, our analysis may be regarded as a pilot study and a first step in quantifying the impact of female underrepresentation on estimates of treatment effect. It lays the groundwork for systematic research within specific CVD domains or drug classes and underscores the need to explore the underlying reasons for female underrepresentation in clinical CVD trials.

## Conclusion

Although females were underrepresented in the selected WCN-CVD trials, their participation was sufficient to reliably exclude large sex differences in treatment efficacy. This finding was made in a limited and heterogeneous group of trials. We therefore emphasize the need for further research within specific disease areas. Moreover, increasing female representation in cardiovascular trials remains essential to ensure fairness and to gain a deeper understanding of sex-based differences in safety.

## Supplementary Information

Table S1. Prevalence data

Table S2. Participation to prevalence ratios of the included trials.

Table S3 A. Characteristics of included event-driven studies.

Table S3 B. Sex-specific REM of included event-driven studies.

Supplemental Fig. 1. Flowchart of the completed cardiovascular trials

Supplemental Fig. 2. Participation to Prevalence Ratios (PPR) per trial category

Supplemental Fig. 3. Forest plot difference log(REM)

**Supplemental Fig. 4. **Funnel plot difference log(REM)
