# Supplementary material for: A comprehensive analysis of female participation in cardiovascular trials involving the WCN investigator network
Source: Neth Heart J. 2025 Nov 12;33(12):404–11. doi: 10.1007/s12471-025-01999-4 (PMC12638513; doi:10.1007/s12471-025-01999-4)
Supplement: Supplementary file 1 — Table S1. Prevalence data [file 12471_2025_1999_MOESM1_ESM.docx]

| **Disease** | **Percentage of females** |
| --- | --- |
| *Dutch population^1^* |  |
| Acute Coronary Syndrome | 38 (1) |
| Acute Myocardial Infarction | 29 (2) |
| Angina pectoris | 45 (2) |
| Atrial Fibrillation | 45 (2) |
| Cardiovascular disease, general | 48 (2) |
| Coronary Heart Disease | 38 (1) |
| Diabetes Mellitus | 47 (3) |
| Heart Failure, both types | 53 (2) |
| Pacemaker / ICD | 42 (2) |
| *Swedish heart failure registry (approximately* *43k patients)* |  |
| HFrEF | 29 (4) |
| HFpEF | 55 (4) |
| *United States, general population (approximately 50k residents)* |  |
| Supraventriculaire tachycardia | 57 (5) |

**^1^** This concerns the Dutch general population data, which makes it impossible to determine the exact number of people involved.

**References:**

1. Volksgezondheid en Zorg. Coronaire hartziekten | Leeftijd en geslacht. [Available from: [https://www.vzinfo.nl/coronaire-hartziekten/leeftijd-en-geslacht#:~:text=In%202021%20waren%20er%20naar,33%2C6%20per%201.000%20vrouwen](https://www.vzinfo.nl/coronaire-hartziekten/leeftijd-en-geslacht#:~:text=In%202021%20waren%20er%20naar,33%2C6%20per%201.000%20vrouwen).

2. Nederlandse Hart Registratie. Hart- en vaatcijfers. 2021 [Available from: <https://www.hartenvaatcijfers.nl/>.

3. Volksgezondheid en Zorg. Diabetes mellitus | Leeftijd en geslacht. [Available from: <https://www.vzinfo.nl/diabetes-mellitus/leeftijd-en-geslacht>.

4. Davide S, Alicia U, Ola V, Anna S, Ulrika Ljung F, Giuseppe MCR, et al. Sex-Based Differences in Heart Failure Across the Ejection Fraction Spectrum. JACC: Heart Failure. 2019;7(6):505-15.

5. Orejarena LA, Vidaillet H, Jr., DeStefano F, Nordstrom DL, Vierkant RA, Smith PN, Hayes JJ. Paroxysmal supraventricular tachycardia in the general population. J Am Coll Cardiol. 1998;31(1):150-7.
