# Supplementary material for: A comprehensive analysis of female participation in cardiovascular trials involving the WCN investigator network
Source: Neth Heart J. 2025 Nov 12;33(12):404–11. doi: 10.1007/s12471-025-01999-4 (PMC12638513; doi:10.1007/s12471-025-01999-4)
Supplement: Supplementary file 2 — Table S2. Participation to prevalence ratios of the included trials. [file 12471_2025_1999_MOESM2_ESM.docx]

| **Trial** | **Percentage of females in the target population** | | **Target population, further defined** | **Percentage of females enrolled in the trial** | **PPR** |
| --- | --- | --- | --- | --- | --- |
| *Target population: cardiac arrhythmia* | | | | | |
| PADIT (1) | 42 | | Pacemaker / ICD | 34 | 0.81 |
| RACE III (2) | 45 | | AF | 21 | 0.47 |
| PIONEER (3) | 45 | | AF | 26 | 0.57 |
| DIONYSOS (4) | 45 | | AF | 29 | 0.65 |
| Re-Ve-Ra 201 (5) | 45 | | AF | 32 | 0.71 |
| ACTIVE (6) | 45 | | AF | 34 | 0.76 |
| RACE II (7) | 45 | | AF | 34 | 0.77 |
| ARISTOTLE (8) | 45 | | AF | 35 | 0.79 |
| Pallas (9) | 45 | | AF | 35 | 0.79 |
| ARTESIA (10) | 45 | | AF | 36 | 0.81 |
| ASTAIRE (11) | 45 | | AF | 36 | 0.81 |
| RE-LY (12) | 45 | | AF | 36 | 0.81 |
| RACE (13) | 45 | | AF | 37 | 0.82 |
| BOREALIS (14) | 45 | | AF | 39 | 0.87 |
| ROCKET AF (15) | 45 | | AF | 40 | 0.89 |
| PACIFIC-AF (16) | 45 | | AF | 41 | 0.92 |
| ATHENA (17) | 45 | | AF | 47 | 1.05 |
| RAPID (18) | 57 | | SVT | 71 | 1.25 |
| *Target population: atherosclerotic cardiovascular disease* | | | | | |
| CLARITY-TIMI 28 (19) | | 29 | STEMI | 20 | 0.68 |
| CAPTORS II (20) | | 29 | AMI | 20 | 0.69 |
| ASSENT-3 (21) | | 29 | AMI | 23 | 0.80 |
| PACIFIC-AMI (22) | | 29 | AMI | 23 | 0.80 |
| PEGASUS-TIMI 54 (23) | | 29 | AMI | 24 | 0.83 |
| PARADISE-MI (24) | | 29 | AMI | 24 | 0.84 |
| EMPACT-MI (25) | | 29 | AMI | 25 | 0.87 |
| CANTOS (26) | | 29 | AMI | 26 | 0.89 |
| AEGIS-II (27) | | 29 | AMI | 26 | 0.90 |
| LATITUDE-TIMI 60 (28) | | 29 | AMI | 30 | 1.03 |
| LoDoCo2 (29) | | 38 | CCD | 15 | 0.40 |
| Stability LPL II (30) | | 38 | CCD | 19 | 0.50 |
| COMPASS (31) | | 38 | CCD | 22 | 0.58 |
| SIGNIFY (32) | | 38 | CCD | 28 | 0.73 |
| AURORA (PET MPI) (33) | | 38 | CAD | 32 | 0.85 |
| Dal-OUTCOMES (34) | | 38 | ACS | 19 | 0.50 |
| RUBY (35) | | 38 | ACS | 21 | 0.54 |
| dal-GenE (36) | | 38 | ACS | 23 | 0.59 |
| ATLAS ACS-TIMI 46 (37) | | 38 | ACS | 23 | 0.59 |
| Dal-ACUTE (38) | | 38 | ACS | 23 | 0.59 |
| Improve-IT (39) | | 38 | ACS | 24 | 0.63 |
| GEMINI-ACS-1 (40) | | 38 | ACS | 25 | 0.65 |
| ODYSSEY OUTCOMES (41) | | 38 | ACS | 25 | 0.65 |
| ATLAS ACS 2-TIMI 51 (42) | | 38 | ACS | 25 | 0.66 |
| SOLID-TIMI 52 (43) | | 38 | ACS | 26 | 0.66 |
| VISTA-16 (44) | | 38 | ACS | 26 | 0.68 |
| ARCHIPELAGO (45) | | 38 | ACS | 26 | 0.68 |
| TRACER (46) | | 38 | ACS | 28 | 0.73 |
| PLATO (47) | | 38 | ACS | 28 | 0.74 |
| AUGUSTUS (48) | | 38 | ACS | 29 | 0.75 |
| APPRAISE-2 (49) | | 38 | ACS | 32 | 0.84 |
| TRILOGY ACS (50) | | 38 | ACS | 39 | 1.02 |
| FEMINA (51) | | 45 | AP | 15 | 0.33 |
| ACTION (52) | | 45 | SAP | 21 | 0.46 |
| ACCELERATE (53) | | 48 | HRCVD | 23 | 0.48 |
| TRA 2P-TIMI 50 (54) | | 48 | HRCVD | 24 | 0.50 |
| FOURIER (55) | | 48 | HRCVD + hypercholestrolemia | 25 | 0.51 |
| SPIRE-1 (56) | | 48 | HRCVD | 26 | 0.55 |
| SELECT (57) | | 48 | CVD + obesity | 28 | 0.58 |
| REDUCE-IT (58) | | 48 | HRCVD + hypertriglyceridemia | 29 | 0.60 |
| SPIRE-2 (56) | | 48 | HRCVD | 34 | 0.72 |
| STRENGTH (59) | | 48 | HRCVD + hypertriglyceridemia | 35 | 0.73 |
| CLEAR Outcomes (60) | | 48 | HRCVD + statin intolerant | 48 | 1.01 |
| *Target population: diabetes mellitus* | | | | | |
| PROMINENT (61) | | 47 | DM2 + HRCVD | 28 | 0.59 |
| EMPA-REG OUTCOME (62) | | 47 | DM2 + CVD | 29 | 0.61 |
| TECOS (63) | | 47 | DM2 + CVD | 29 | 0.63 |
| Harmony Outcomes (64) | | 47 | DM2 + CVD | 31 | 0.66 |
| ELIXA (65) | | 47 | DM2 + AMI/UA | 31 | 0.66 |
| THEMIS (66) | | 47 | DM2 + CCD | 31 | 0.67 |
| SAVOR-TIMI 53 (67) | | 47 | DM2 + (HR)CVD | 33 | 0.71 |
| ORIGIN (68) | | 47 | DM2 + HRCVD | 35 | 0.75 |
| DECLARE-TIMI 58 (69) | | 47 | DM2 + HRCVD | 37 | 0.80 |
| EXSCEL (70) | | 47 | DM2 | 38 | 0.82 |
| BEST (71) | | 47 | DM2 | 38 | 0.82 |
| CAROLINA (72) | | 47 | DM2 + HRCVD | 40 | 0.86 |
| TIDE (73) | 47 | | DM2 + HRCVD | 41 | 0.88 |
| OMNEON (74) | 47 | | DM2 | 44 | 0.93 |
| *Target population: heart failure* | | | | | |
| METEORIC-HF (75) | 29 | | HFrEF | 15 | 0.52 |
| BEAUTIFUL (76) | 29 | | HFrEF | 17 | 0.59 |
| GENETIC-AF (77) | 29 | | HFrEF + AF | 18 | 0.62 |
| SOCRATES-REDUCED (78) | 29 | | HFrEF | 20 | 0.68 |
| WARCEF (79) | 29 | | HFrEF | 20 | 0.69 |
| GALACTIC-HF (80) | 29 | | HFrEF | 21 | 0.73 |
| OUTSTEP-HF (81) | 29 | | HFrEF | 21 | 0.74 |
| ATMOSPHERE (82) | 29 | | HFrEF | 22 | 0.75 |
| PARADIGM-HF (83) | 29 | | HFrEF | 22 | 0.75 |
| EMPHASIS-HF (84) | 29 | | HFrEF | 22 | 0.77 |
| STAND-UP AHF (85) | 29 | | HFrEF + AHF | 22 | 0.78 |
| ARTS-HF (86) | 29 | | HFrEF | 23 | 0.78 |
| COMMANDER (87) | 29 | | HFrEF | 23 | 0.79 |
| DAPA-HF (88) | 29 | | HFrEF | 23 | 0.81 |
| CORONA (89) | 29 | | HFrEF | 24 | 0.81 |
| SHIFT (90) | 29 | | HFrEF | 24 | 0.81 |
| VICTORIA (91) | 29 | | HFrEF | 24 | 0.82 |
| EMPEROR-Reduced (92) | 29 | | HFrEF | 24 | 0.83 |
| PROGRESS (93) | 29 | | HFrEF | 24 | 0.83 |
| DIAMOND (94) | 29 | | HFrEF | 27 | 0.93 |
| CIBIS III (95) | 29 | | HFrEF | 32 | 1.10 |
| CHARM-Alternative (96) | 29 | | HFrEF + intolerant to ACE inhibitors | 32 | 1.10 |
| RED-HF (97) | 29 | | HFrEF | 41 | 1.43 |
| LIK066 (98) | 53 | | CHF | 28 | 0.53 |
| EMPA-RESPONSE-AHF (99) | 53 | | AHF | 33 | 0.62 |
| SOLOIST-WHF (100) | 53 | | AHF + DM2 | 34 | 0.64 |
| EMPULSE (101) | 53 | | AHF | 34 | 0.64 |
| ASCEND-HF (102) | 53 | | AHF | 34 | 0.65 |
| TRUE-AHF (103) | 53 | | AHF | 34 | 0.65 |
| RELAX-AHF-2 (104) | 53 | | AHF | 40 | 0.76 |
| AFFIRM-AHF (105) | 53 | | AHF | 45 | 0.84 |
| DELIVER (106) | 55 | | HFmrEF / HFpEF | 44 | 0.80 |
| EMPEROR-Preserved (107) | 55 | | HFpEF | 45 | 0.81 |
| STEP-HFpEF DM (108) | 55 | | HFpEF + DM2 | 44 | 0.81 |
| SOCRATES-PRESERVED (109) | 55 | | HFpEF | 48 | 0.87 |
| PARALLAX (110) | 55 | | HFpEF | 51 | 0.92 |
| PARAGON-HF (111) | 55 | | HFpEF | 52 | 0.94 |
| STEP-HFpEF (112) | 55 | | HFpEF | 56 | 1.02 |
| PARAMOUNT (113) | 55 | | HFpEF | 56 | 1.03 |
| EDIFY (114) | 55 | | HFpEF | 65 | 1.18 |

Abbreviations used: ICD = implantable cardioverter-defibrillator; AF = atrial fibrillation; SVT = supraventricular tachycardia; STEMI = ST-elevation myocardial infarction; AMI = acute myocardial infarction; CCD = chronic coronary disease; CAD = coronary artery disease; ACS = acute coronary syndrome; AP = anginta pectoris; SAP = stable angina pectoris; HRCVD = high risk cardiovascular disease; CVD = cardiovascular disease; DM2 = diabetes mellitus type 2; UA = unstable angina; HFrEF = heart failure with reduced ejection fraction; AHF = acute heart failure; HFmrEF = heart failure with mid-range ejection fraction; HFpEF = heart failure with preserved ejection fraction.

**References**

1. Krahn AD, Longtin Y, Philippon F, Birnie DH, Manlucu J, Angaran P, et al. Prevention of Arrhythmia Device Infection Trial: The PADIT Trial. J Am Coll Cardiol. 2018;72(24):3098-109.

2. Rienstra M, Hobbelt AH, Alings M, Tijssen JGP, Smit MD, Brügemann J, et al. Targeted therapy of underlying conditions improves sinus rhythm maintenance in patients with persistent atrial fibrillation: results of the RACE 3 trial. European Heart Journal. 2018;39(32):2987-96.

3. Gibson CM, Mehran R, Bode C, Halperin J, Verheugt FW, Wildgoose P, et al. Prevention of Bleeding in Patients with Atrial Fibrillation Undergoing PCI. New England Journal of Medicine. 2016;375(25):2423-34.

4. Le Heuzey JY, De Ferrari GM, Radzik D, Santini M, Zhu J, Davy JM. A short-term, randomized, double-blind, parallel-group study to evaluate the efficacy and safety of dronedarone versus amiodarone in patients with persistent atrial fibrillation: the DIONYSOS study. J Cardiovasc Electrophysiol. 2010;21(6):597-605.

5. Camm AJ, Piccini JP, Alings M, Dorian P, Gosselin G, Guertin MC, et al. Multicenter, Phase 2, Randomized Controlled Study of the Efficacy and Safety of Etripamil Nasal Spray for the Acute Reduction of Rapid Ventricular Rate in Patients With Symptomatic Atrial Fibrillation (ReVeRA-201). Circ Arrhythm Electrophysiol. 2023;16(12):639-50.

6. Active Writing Group of the ACTIVE Investigators, Connolly S, Pogue J, Hart R, Pfeffer M, Hohnloser S, et al. Clopidogrel plus aspirin versus oral anticoagulation for atrial fibrillation in the Atrial fibrillation Clopidogrel Trial with Irbesartan for prevention of Vascular Events (ACTIVE W): a randomised controlled trial. Lancet. 2006;367(9526):1903-12.

7. Van Gelder IC, Groenveld HF, Crijns HJ, Tuininga YS, Tijssen JG, Alings AM, et al. Lenient versus strict rate control in patients with atrial fibrillation. N Engl J Med. 2010;362(15):1363-73.

8. Granger CB, Alexander JH, McMurray JJ, Lopes RD, Hylek EM, Hanna M, et al. Apixaban versus warfarin in patients with atrial fibrillation. N Engl J Med. 2011;365(11):981-92.

9. Connolly SJ, Camm AJ, Halperin JL, Joyner C, Alings M, Amerena J, et al. Dronedarone in high-risk permanent atrial fibrillation. N Engl J Med. 2011;365(24):2268-76.

10. Healey JS, Lopes RD, Granger CB, Alings M, Rivard L, McIntyre WF, et al. Apixaban for Stroke Prevention in Subclinical Atrial Fibrillation. N Engl J Med. 2024;390(2):107-17.

11. Rónaszéki A, Alings M, Egstrup K, Gaciong Z, Hranai M, Király C, et al. Pharmacological cardioversion of atrial fibrillation—a double-blind, randomized, placebo-controlled, multicentre, dose-escalation study of AZD1305 given intravenously. EP Europace. 2011;13(8):1148-56.

12. Connolly SJ, Ezekowitz MD, Yusuf S, Eikelboom J, Oldgren J, Parekh A, et al. Dabigatran versus warfarin in patients with atrial fibrillation. N Engl J Med. 2009;361(12):1139-51.

13. Van Gelder IC, Hagens VE, Bosker HA, Kingma JH, Kamp O, Kingma T, et al. A Comparison of Rate Control and Rhythm Control in Patients with Recurrent Persistent Atrial Fibrillation. New England Journal of Medicine. 2002;347(23):1834-40.

14. Buller HR, Halperin J, Hankey GJ, Pillion G, Prins MH, Raskob GE. Comparison of idrabiotaparinux with vitamin K antagonists for prevention of thromboembolism in patients with atrial fibrillation: the Borealis-Atrial Fibrillation Study. J Thromb Haemost. 2014;12(6):824-30.

15. Patel MR, Mahaffey KW, Garg J, Pan G, Singer DE, Hacke W, et al. Rivaroxaban versus warfarin in nonvalvular atrial fibrillation. N Engl J Med. 2011;365(10):883-91.

16. Piccini JP, Caso V, Connolly SJ, Fox KAA, Oldgren J, Jones WS, et al. Safety of the oral factor XIa inhibitor asundexian compared with apixaban in patients with atrial fibrillation (PACIFIC-AF): a multicentre, randomised, double-blind, double-dummy, dose-finding phase 2 study. Lancet. 2022;399(10333):1383-90.

17. Hohnloser SH, Crijns HJ, van Eickels M, Gaudin C, Page RL, Torp-Pedersen C, et al. Effect of dronedarone on cardiovascular events in atrial fibrillation. N Engl J Med. 2009;360(7):668-78.

18. Stambler BS, Camm AJ, Alings M, Dorian P, Heidbuchel H, Houtgraaf J, et al. Self-administered intranasal etripamil using a symptom-prompted, repeat-dose regimen for atrioventricular-nodal-dependent supraventricular tachycardia (RAPID): a multicentre, randomised trial. Lancet. 2023;402(10396):118-28.

19. Sabatine MS, Cannon CP, Gibson CM, López-Sendón JL, Montalescot G, Theroux P, et al. Addition of clopidogrel to aspirin and fibrinolytic therapy for myocardial infarction with ST-segment elevation. N Engl J Med. 2005;352(12):1179-89.

20. Armstrong PW, Burton J, Pakola S, Molhoek PG, Betriu A, Tendera M, et al. Collaborative Angiographic Patency Trial Of Recombinant Staphylokinase (CAPTORS II). Am Heart J. 2003;146(3):484-8.

21. Assessment of the Safety and Efficacy of a New Thrombolytic Regimen (ASSENT)-3 Investigators. Efficacy and safety of tenecteplase in combination with enoxaparin, abciximab, or unfractionated heparin: the ASSENT-3 randomised trial in acute myocardial infarction. Lancet. 2001;358(9282):605-13.

22. Rao SV, Kirsch B, Bhatt DL, Budaj A, Coppolecchia R, Eikelboom J, et al. A Multicenter, Phase 2, Randomized, Placebo-Controlled, Double-Blind, Parallel-Group, Dose-Finding Trial of the Oral Factor XIa Inhibitor Asundexian to Prevent Adverse Cardiovascular Outcomes After Acute Myocardial Infarction. Circulation. 2022;146(16):1196-206.

23. Bonaca MP, Bhatt DL, Cohen M, Steg PG, Storey RF, Jensen EC, et al. Long-term use of ticagrelor in patients with prior myocardial infarction. N Engl J Med. 2015;372(19):1791-800.

24. Pfeffer MA, Claggett B, Lewis EF, Granger CB, Køber L, Maggioni AP, et al. Angiotensin Receptor-Neprilysin Inhibition in Acute Myocardial Infarction. N Engl J Med. 2021;385(20):1845-55.

25. Butler J, Jones WS, Udell JA, Anker SD, Petrie MC, Harrington J, et al. Empagliflozin after Acute Myocardial Infarction. N Engl J Med. 2024;390(16):1455-66.

26. Ridker PM, Everett BM, Thuren T, MacFadyen JG, Chang WH, Ballantyne C, et al. Antiinflammatory Therapy with Canakinumab for Atherosclerotic Disease. N Engl J Med. 2017;377(12):1119-31.

27. Gibson CM, Duffy D, Korjian S, Bahit MC, Chi G, Alexander JH, et al. Apolipoprotein A1 Infusions and Cardiovascular Outcomes after Acute Myocardial Infarction. N Engl J Med. 2024;390(17):1560-71.

28. O'Donoghue ML, Glaser R, Cavender MA, Aylward PE, Bonaca MP, Budaj A, et al. Effect of Losmapimod on Cardiovascular Outcomes in Patients Hospitalized With Acute Myocardial Infarction: A Randomized Clinical Trial. Jama. 2016;315(15):1591-9.

29. Nidorf SM, Fiolet ATL, Mosterd A, Eikelboom JW, Schut A, Opstal TSJ, et al. Colchicine in Patients with Chronic Coronary Disease. N Engl J Med. 2020;383(19):1838-47.

30. The STABILITY Investigators, White HD, Held C, Stewart R, Tarka E, Brown R, et al. Darapladib for preventing ischemic events in stable coronary heart disease. N Engl J Med. 2014;370(18):1702-11.

31. Eikelboom JW, Connolly SJ, Bosch J, Dagenais GR, Hart RG, Shestakovska O, et al. Rivaroxaban with or without Aspirin in Stable Cardiovascular Disease. New England Journal of Medicine. 2017;377(14):1319-30.

32. Fox K, Ford I, Steg PG, Tardif JC, Tendera M, Ferrari R, Investigators S. Ivabradine in stable coronary artery disease without clinical heart failure. N Engl J Med. 2014;371(12):1091-9.

33. Maddahi J, Agostini D, Bateman TM, Bax JJ, Beanlands RSB, Berman DS, et al. Flurpiridaz F-18 PET Myocardial Perfusion Imaging in Patients With Suspected Coronary Artery Disease. J Am Coll Cardiol. 2023;82(16):1598-610.

34. Schwartz GG, Olsson AG, Abt M, Ballantyne CM, Barter PJ, Brumm J, et al. Effects of dalcetrapib in patients with a recent acute coronary syndrome. N Engl J Med. 2012;367(22):2089-99.

35. Bartorelli AL, Koh TH, Di Pede F, Reimers B, Thuesen L, Amann FW, et al. Distal embolic protection during percutaneous coronary intervention in patients with acute coronary syndromes: the RUBY study. Acute Card Care. 2006;8(3):148-54.

36. Tardif JC, Pfeffer MA, Kouz S, Koenig W, Maggioni AP, McMurray JJV, et al. Pharmacogenetics-guided dalcetrapib therapy after an acute coronary syndrome: the dal-GenE trial. Eur Heart J. 2022;43(39):3947-56.

37. Mega JL, Braunwald E, Mohanavelu S, Burton P, Poulter R, Misselwitz F, et al. Rivaroxaban versus placebo in patients with acute coronary syndromes (ATLAS ACS-TIMI 46): a randomised, double-blind, phase II trial. Lancet. 2009;374(9683):29-38.

38. Ray KK, Ditmarsch M, Kallend D, Niesor EJ, Suchankova G, Upmanyu R, et al. The effect of cholesteryl ester transfer protein inhibition on lipids, lipoproteins, and markers of HDL function after an acute coronary syndrome: the dal-ACUTE randomized trial. Eur Heart J. 2014;35(27):1792-800.

39. Cannon CP, Blazing MA, Giugliano RP, McCagg A, White JA, Theroux P, et al. Ezetimibe Added to Statin Therapy after Acute Coronary Syndromes. N Engl J Med. 2015;372(25):2387-97.

40. Ohman EM, Roe MT, Steg PG, James SK, Povsic TJ, White J, et al. Clinically significant bleeding with low-dose rivaroxaban versus aspirin, in addition to P2Y12 inhibition, in acute coronary syndromes (GEMINI-ACS-1): a double-blind, multicentre, randomised trial. Lancet. 2017;389(10081):1799-808.

41. Schwartz GG, Steg PG, Szarek M, Bhatt DL, Bittner VA, Diaz R, et al. Alirocumab and Cardiovascular Outcomes after Acute Coronary Syndrome. N Engl J Med. 2018;379(22):2097-107.

42. Mega JL, Braunwald E, Wiviott SD, Bassand JP, Bhatt DL, Bode C, et al. Rivaroxaban in patients with a recent acute coronary syndrome. N Engl J Med. 2012;366(1):9-19.

43. O'Donoghue ML, Braunwald E, White HD, Lukas MA, Tarka E, Steg PG, et al. Effect of darapladib on major coronary events after an acute coronary syndrome: the SOLID-TIMI 52 randomized clinical trial. Jama. 2014;312(10):1006-15.

44. Nicholls SJ, Kastelein JJ, Schwartz GG, Bash D, Rosenson RS, Cavender MA, et al. Varespladib and cardiovascular events in patients with an acute coronary syndrome: the VISTA-16 randomized clinical trial. Jama. 2014;311(3):252-62.

45. Montalescot G, Drexler H, Gallo R, Pearson T, Thoenes M, Bhatt DL. Effect of irbesartan and enalapril in non-ST elevation acute coronary syndrome: results of the randomized, double-blind ARCHIPELAGO study. Eur Heart J. 2009;30(22):2733-41.

46. Tricoci P, Huang Z, Held C, Moliterno DJ, Armstrong PW, Van de Werf F, et al. Thrombin-receptor antagonist vorapaxar in acute coronary syndromes. N Engl J Med. 2012;366(1):20-33.

47. Wallentin L, Becker RC, Budaj A, Cannon CP, Emanuelsson H, Held C, et al. Ticagrelor versus clopidogrel in patients with acute coronary syndromes. N Engl J Med. 2009;361(11):1045-57.

48. Lopes RD, Heizer G, Aronson R, Vora AN, Massaro T, Mehran R, et al. Antithrombotic Therapy after Acute Coronary Syndrome or PCI in Atrial Fibrillation. N Engl J Med. 2019;380(16):1509-24.

49. Alexander JH, Lopes RD, James S, Kilaru R, He Y, Mohan P, et al. Apixaban with antiplatelet therapy after acute coronary syndrome. N Engl J Med. 2011;365(8):699-708.

50. Roe MT, Armstrong PW, Fox KA, White HD, Prabhakaran D, Goodman SG, et al. Prasugrel versus clopidogrel for acute coronary syndromes without revascularization. N Engl J Med. 2012;367(14):1297-309.

51. Dunselman P, Liem AH, Verdel G, Kragten H, Bosma A, Bernink P. Addition of felodipine to metoprolol vs replacement of metoprolol by felodipine in patients with angina pectoris despite adequate beta-blockade. Results of the Felodipine ER and Metoprolol CR in Angina (FEMINA) Study. Working Group on Cardiovascular Research, The Netherlands (WCN). Eur Heart J. 1997;18(11):1755-64.

52. Poole-Wilson PA, Lubsen J, Kirwan BA, van Dalen FJ, Wagener G, Danchin N, et al. Effect of long-acting nifedipine on mortality and cardiovascular morbidity in patients with stable angina requiring treatment (ACTION trial): randomised controlled trial. Lancet. 2004;364(9437):849-57.

53. Lincoff AM, Nicholls SJ, Riesmeyer JS, Barter PJ, Brewer HB, Fox KAA, et al. Evacetrapib and Cardiovascular Outcomes in High-Risk Vascular Disease. N Engl J Med. 2017;376(20):1933-42.

54. Morrow DA, Braunwald E, Bonaca MP, Ameriso SF, Dalby AJ, Fish MP, et al. Vorapaxar in the secondary prevention of atherothrombotic events. N Engl J Med. 2012;366(15):1404-13.

55. Sabatine MS, Giugliano RP, Keech AC, Honarpour N, Wiviott SD, Murphy SA, et al. Evolocumab and Clinical Outcomes in Patients with Cardiovascular Disease. N Engl J Med. 2017;376(18):1713-22.

56. Ridker PM, Revkin J, Amarenco P, Brunell R, Curto M, Civeira F, et al. Cardiovascular Efficacy and Safety of Bococizumab in High-Risk Patients. N Engl J Med. 2017;376(16):1527-39.

57. Lincoff AM, Brown-Frandsen K, Colhoun HM, Deanfield J, Emerson SS, Esbjerg S, et al. Semaglutide and Cardiovascular Outcomes in Obesity without Diabetes. N Engl J Med. 2023;389(24):2221-32.

58. Bhatt DL, Steg PG, Miller M, Brinton EA, Jacobson TA, Ketchum SB, et al. Cardiovascular Risk Reduction with Icosapent Ethyl for Hypertriglyceridemia. N Engl J Med. 2019;380(1):11-22.

59. Nicholls SJ, Lincoff AM, Garcia M, Bash D, Ballantyne CM, Barter PJ, et al. Effect of High-Dose Omega-3 Fatty Acids vs Corn Oil on Major Adverse Cardiovascular Events in Patients at High Cardiovascular Risk: The STRENGTH Randomized Clinical Trial. Jama. 2020;324(22):2268-80.

60. Nissen SE, Lincoff AM, Brennan D, Ray KK, Mason D, Kastelein JJP, et al. Bempedoic Acid and Cardiovascular Outcomes in Statin-Intolerant Patients. N Engl J Med. 2023;388(15):1353-64.

61. Das Pradhan A, Glynn RJ, Fruchart JC, MacFadyen JG, Zaharris ES, Everett BM, et al. Triglyceride Lowering with Pemafibrate to Reduce Cardiovascular Risk. N Engl J Med. 2022;387(21):1923-34.

62. Zinman B, Wanner C, Lachin JM, Fitchett D, Bluhmki E, Hantel S, et al. Empagliflozin, Cardiovascular Outcomes, and Mortality in Type 2 Diabetes. N Engl J Med. 2015;373(22):2117-28.

63. Green JB, Bethel MA, Armstrong PW, Buse JB, Engel SS, Garg J, et al. Effect of Sitagliptin on Cardiovascular Outcomes in Type 2 Diabetes. N Engl J Med. 2015;373(3):232-42.

64. Hernandez AF, Green JB, Janmohamed S, D'Agostino RB, Sr., Granger CB, Jones NP, et al. Albiglutide and cardiovascular outcomes in patients with type 2 diabetes and cardiovascular disease (Harmony Outcomes): a double-blind, randomised placebo-controlled trial. Lancet. 2018;392(10157):1519-29.

65. Pfeffer MA, Claggett B, Diaz R, Dickstein K, Gerstein HC, Køber LV, et al. Lixisenatide in Patients with Type 2 Diabetes and Acute Coronary Syndrome. N Engl J Med. 2015;373(23):2247-57.

66. Steg PG, Bhatt DL, Simon T, Fox K, Mehta SR, Harrington RA, et al. Ticagrelor in Patients with Stable Coronary Disease and Diabetes. N Engl J Med. 2019;381(14):1309-20.

67. Scirica BM, Bhatt DL, Braunwald E, Steg PG, Davidson J, Hirshberg B, et al. Saxagliptin and cardiovascular outcomes in patients with type 2 diabetes mellitus. N Engl J Med. 2013;369(14):1317-26.

68. Investigators OT, Gerstein HC, Bosch J, Dagenais GR, Díaz R, Jung H, et al. Basal insulin and cardiovascular and other outcomes in dysglycemia. N Engl J Med. 2012;367(4):319-28.

69. Wiviott SD, Raz I, Bonaca MP, Mosenzon O, Kato ET, Cahn A, et al. Dapagliflozin and Cardiovascular Outcomes in Type 2 Diabetes. N Engl J Med. 2019;380(4):347-57.

70. Holman RR, Bethel MA, Mentz RJ, Thompson VP, Lokhnygina Y, Buse JB, et al. Effects of Once-Weekly Exenatide on Cardiovascular Outcomes in Type 2 Diabetes. N Engl J Med. 2017;377(13):1228-39.

71. Halvorsen YD, Walford G, Thurber T, Russell H, Massaro M, Freeman MW. A 12-week, randomized, double-blind, placebo-controlled, four-arm dose-finding phase 2 study evaluating bexagliflozin as monotherapy for adults with type 2 diabetes. Diabetes Obes Metab. 2020;22(4):566-73.

72. Rosenstock J, Kahn SE, Johansen OE, Zinman B, Espeland MA, Woerle HJ, et al. Effect of Linagliptin vs Glimepiride on Major Adverse Cardiovascular Outcomes in Patients With Type 2 Diabetes: The CAROLINA Randomized Clinical Trial. Jama. 2019;322(12):1155-66.

73. Punthakee Z, Bosch J, Dagenais G, Diaz R, Holman R, Probstfield J, et al. Design, history and results of the Thiazolidinedione Intervention with vitamin D Evaluation (TIDE) randomised controlled trial. Diabetologia. 2012;55(1):36-45.

74. Sheu WH, Gantz I, Chen M, Suryawanshi S, Mirza A, Goldstein BJ, et al. Safety and Efficacy of Omarigliptin (MK-3102), a Novel Once-Weekly DPP-4 Inhibitor for the Treatment of Patients With Type 2 Diabetes. Diabetes Care. 2015;38(11):2106-14.

75. Lewis GD, Voors AA, Cohen-Solal A, Metra M, Whellan DJ, Ezekowitz JA, et al. Effect of Omecamtiv Mecarbil on Exercise Capacity in Chronic Heart Failure With Reduced Ejection Fraction: The METEORIC-HF Randomized Clinical Trial. Jama. 2022;328(3):259-69.

76. Fox K, Ford I, Steg PG, Tendera M, Ferrari R. Ivabradine for patients with stable coronary artery disease and left-ventricular systolic dysfunction (BEAUTIFUL): a randomised, double-blind, placebo-controlled trial. Lancet. 2008;372(9641):807-16.

77. Piccini JP, Abraham WT, Dufton C, Carroll IA, Healey JS, van Veldhuisen DJ, et al. Bucindolol for the Maintenance of Sinus Rhythm in a Genotype-Defined HF Population: The GENETIC-AF Trial. JACC Heart Fail. 2019;7(7):586-98.

78. Gheorghiade M, Greene SJ, Butler J, Filippatos G, Lam CS, Maggioni AP, et al. Effect of Vericiguat, a Soluble Guanylate Cyclase Stimulator, on Natriuretic Peptide Levels in Patients With Worsening Chronic Heart Failure and Reduced Ejection Fraction: The SOCRATES-REDUCED Randomized Trial. Jama. 2015;314(21):2251-62.

79. Homma S, Thompson JL, Pullicino PM, Levin B, Freudenberger RS, Teerlink JR, et al. Warfarin and aspirin in patients with heart failure and sinus rhythm. N Engl J Med. 2012;366(20):1859-69.

80. Teerlink JR, Diaz R, Felker GM, McMurray JJV, Metra M, Solomon SD, et al. Cardiac Myosin Activation with Omecamtiv Mecarbil in Systolic Heart Failure. N Engl J Med. 2021;384(2):105-16.

81. Piepoli MF, Hussain RI, Comin-Colet J, Dosantos R, Ferber P, Jaarsma T, Edelmann F. OUTSTEP-HF: randomised controlled trial comparing short-term effects of sacubitril/valsartan versus enalapril on daily physical activity in patients with chronic heart failure with reduced ejection fraction. Eur J Heart Fail. 2021;23(1):127-35.

82. McMurray JJ, Krum H, Abraham WT, Dickstein K, Kober LV, Desai AS, et al. Aliskiren, Enalapril, or Aliskiren and Enalapril in Heart Failure. N Engl J Med. 2016;374(16):1521-32.

83. McMurray JJ, Packer M, Desai AS, Gong J, Lefkowitz MP, Rizkala AR, et al. Angiotensin-neprilysin inhibition versus enalapril in heart failure. N Engl J Med. 2014;371(11):993-1004.

84. Zannad F, McMurray JJ, Krum H, van Veldhuisen DJ, Swedberg K, Shi H, et al. Eplerenone in patients with systolic heart failure and mild symptoms. N Engl J Med. 2011;364(1):11-21.

85. Felker GM, McMurray JJV, Cleland JG, O'Connor CM, Teerlink JR, Voors AA, et al. Effects of a Novel Nitroxyl Donor in Acute Heart Failure: The STAND-UP AHF Study. JACC Heart Fail. 2021;9(2):146-57.

86. Filippatos G, Anker SD, Böhm M, Gheorghiade M, Køber L, Krum H, et al. A randomized controlled study of finerenone vs. eplerenone in patients with worsening chronic heart failure and diabetes mellitus and/or chronic kidney disease. Eur Heart J. 2016;37(27):2105-14.

87. Zannad F, Anker SD, Byra WM, Cleland JGF, Fu M, Gheorghiade M, et al. Rivaroxaban in Patients with Heart Failure, Sinus Rhythm, and Coronary Disease. N Engl J Med. 2018;379(14):1332-42.

88. McMurray JJV, Solomon SD, Inzucchi SE, Køber L, Kosiborod MN, Martinez FA, et al. Dapagliflozin in Patients with Heart Failure and Reduced Ejection Fraction. N Engl J Med. 2019;381(21):1995-2008.

89. Kjekshus J, Apetrei E, Barrios V, Böhm M, Cleland JG, Cornel JH, et al. Rosuvastatin in older patients with systolic heart failure. N Engl J Med. 2007;357(22):2248-61.

90. Swedberg K, Komajda M, Böhm M, Borer JS, Ford I, Dubost-Brama A, et al. Ivabradine and outcomes in chronic heart failure (SHIFT): a randomised placebo-controlled study. Lancet. 2010;376(9744):875-85.

91. Armstrong PW, Pieske B, Anstrom KJ, Ezekowitz J, Hernandez AF, Butler J, et al. Vericiguat in Patients with Heart Failure and Reduced Ejection Fraction. N Engl J Med. 2020;382(20):1883-93.

92. Packer M, Anker SD, Butler J, Filippatos G, Pocock SJ, Carson P, et al. Cardiovascular and Renal Outcomes with Empagliflozin in Heart Failure. N Engl J Med. 2020;383(15):1413-24.

93. Butler J, Khan MS, Anker SD, Fonarow GC, Kim RJ, Nodari S, et al. Effects of Elamipretide on Left Ventricular Function in Patients With Heart Failure With Reduced Ejection Fraction: The PROGRESS-HF Phase 2 Trial. J Card Fail. 2020;26(5):429-37.

94. Butler J, Anker SD, Lund LH, Coats AJS, Filippatos G, Siddiqi TJ, et al. Patiromer for the management of hyperkalemia in heart failure with reduced ejection fraction: the DIAMOND trial. Eur Heart J. 2022;43(41):4362-73.

95. Willenheimer R, Van Veldhuisen DJ, Silke B, Erdmann E, Follath F, Krum H, et al. Effect on survival and hospitalization of initiating treatment for chronic heart failure with bisoprolol followed by enalapril, as compared with the opposite sequence: Results of the Randomized Cardiac Insufficiency Bisoprolol Study (CIBIS) III. Circulation. 2005;112(16):2426-35.

96. Granger CB, McMurray JJ, Yusuf S, Held P, Michelson EL, Olofsson B, et al. Effects of candesartan in patients with chronic heart failure and reduced left-ventricular systolic function intolerant to angiotensin-converting-enzyme inhibitors: the CHARM-Alternative trial. Lancet. 2003;362(9386):772-6.

97. Swedberg K, Young JB, Anand IS, Cheng S, Desai AS, Diaz R, et al. Treatment of anemia with darbepoetin alfa in systolic heart failure. N Engl J Med. 2013;368(13):1210-9.

98. de Boer RA, Núñez J, Kozlovski P, Wang Y, Proot P, Keefe D. Effects of the dual sodium-glucose linked transporter inhibitor, licogliflozin vs placebo or empagliflozin in patients with type 2 diabetes and heart failure. Br J Clin Pharmacol. 2020;86(7):1346-56.

99. Damman K, Beusekamp JC, Boorsma EM, Swart HP, Smilde TDJ, Elvan A, et al. Randomized, double-blind, placebo-controlled, multicentre pilot study on the effects of empagliflozin on clinical outcomes in patients with acute decompensated heart failure (EMPA-RESPONSE-AHF). Eur J Heart Fail. 2020;22(4):713-22.

100. Bhatt DL, Szarek M, Steg PG, Cannon CP, Leiter LA, McGuire DK, et al. Sotagliflozin in Patients with Diabetes and Recent Worsening Heart Failure. N Engl J Med. 2021;384(2):117-28.

101. Voors AA, Angermann CE, Teerlink JR, Collins SP, Kosiborod M, Biegus J, et al. The SGLT2 inhibitor empagliflozin in patients hospitalized for acute heart failure: a multinational randomized trial. Nat Med. 2022;28(3):568-74.

102. O'Connor CM, Starling RC, Hernandez AF, Armstrong PW, Dickstein K, Hasselblad V, et al. Effect of nesiritide in patients with acute decompensated heart failure. N Engl J Med. 2011;365(1):32-43.

103. Packer M, O'Connor C, McMurray JJV, Wittes J, Abraham WT, Anker SD, et al. Effect of Ularitide on Cardiovascular Mortality in Acute Heart Failure. N Engl J Med. 2017;376(20):1956-64.

104. Metra M, Teerlink JR, Cotter G, Davison BA, Felker GM, Filippatos G, et al. Effects of Serelaxin in Patients with Acute Heart Failure. N Engl J Med. 2019;381(8):716-26.

105. Ponikowski P, Kirwan BA, Anker SD, McDonagh T, Dorobantu M, Drozdz J, et al. Ferric carboxymaltose for iron deficiency at discharge after acute heart failure: a multicentre, double-blind, randomised, controlled trial. Lancet. 2020;396(10266):1895-904.

106. Solomon SD, McMurray JJV, Claggett B, de Boer RA, DeMets D, Hernandez AF, et al. Dapagliflozin in Heart Failure with Mildly Reduced or Preserved Ejection Fraction. N Engl J Med. 2022;387(12):1089-98.

107. Anker SD, Butler J, Filippatos G, Ferreira JP, Bocchi E, Böhm M, et al. Empagliflozin in Heart Failure with a Preserved Ejection Fraction. N Engl J Med. 2021;385(16):1451-61.

108. Kosiborod MN, Petrie MC, Borlaug BA, Butler J, Davies MJ, Hovingh GK, et al. Semaglutide in Patients with Obesity-Related Heart Failure and Type 2 Diabetes. N Engl J Med. 2024;390(15):1394-407.

109. Pieske B, Maggioni AP, Lam CSP, Pieske-Kraigher E, Filippatos G, Butler J, et al. Vericiguat in patients with worsening chronic heart failure and preserved ejection fraction: results of the SOluble guanylate Cyclase stimulatoR in heArT failurE patientS with PRESERVED EF (SOCRATES-PRESERVED) study. Eur Heart J. 2017;38(15):1119-27.

110. Pieske B, Wachter R, Shah SJ, Baldridge A, Szeczoedy P, Ibram G, et al. Effect of Sacubitril/Valsartan vs Standard Medical Therapies on Plasma NT-proBNP Concentration and Submaximal Exercise Capacity in Patients With Heart Failure and Preserved Ejection Fraction: The PARALLAX Randomized Clinical Trial. Jama. 2021;326(19):1919-29.

111. Solomon SD, McMurray JJV, Anand IS, Ge J, Lam CSP, Maggioni AP, et al. Angiotensin-Neprilysin Inhibition in Heart Failure with Preserved Ejection Fraction. N Engl J Med. 2019;381(17):1609-20.

112. Kosiborod MN, Abildstrøm SZ, Borlaug BA, Butler J, Rasmussen S, Davies M, et al. Semaglutide in Patients with Heart Failure with Preserved Ejection Fraction and Obesity. N Engl J Med. 2023;389(12):1069-84.

113. Solomon SD, Zile M, Pieske B, Voors A, Shah A, Kraigher-Krainer E, et al. The angiotensin receptor neprilysin inhibitor LCZ696 in heart failure with preserved ejection fraction: a phase 2 double-blind randomised controlled trial. Lancet. 2012;380(9851):1387-95.

114. Komajda M, Isnard R, Cohen-Solal A, Metra M, Pieske B, Ponikowski P, et al. Effect of ivabradine in patients with heart failure with preserved ejection fraction: the EDIFY randomized placebo-controlled trial. Eur J Heart Fail. 2017;19(11):1495-503.
