# Supplementary material for: A comprehensive analysis of female participation in cardiovascular trials involving the WCN investigator network
Source: Neth Heart J. 2025 Nov 12;33(12):404–11. doi: 10.1007/s12471-025-01999-4 (PMC12638513; doi:10.1007/s12471-025-01999-4)
Supplement: Supplementary file 3 — Table S3 A. Characteristics of included event-driven studies. [file 12471_2025_1999_MOESM3_ESM.jpg]

| **Trial** | **Patients** |  | **Randomly allocated treatment** | | |  | **Primary efficacy endpoint** |
| --- | --- | --- | --- | --- | --- | --- | --- |
|  |  |  | **Experimental** | **Control** | **Follow-up** |  |  |
| ACCELERATE (1) | HRCVD |  | Evacetrapib | Placebo | 28 months |  | CV death, MI, stroke, coronary revascularization, or hospitalization for UA. |
| ACTION (2) | SAP |  | Nifedipine | Placebo | 4.9 years |  | Death, MI, refractory angina, new overt HF, debilitating stroke, and peripheral revascularization. |
| AEGIS-II (3) | AMI |  | CSL112 | Placebo | 1 year |  | CV death through 90 days follow-up, MI or stroke. |
| AFFIRM-AHF (4) | AHF |  | Ferric carboxymaltose | Placebo | 1 year |  | Total hospitalizations for HF and CV death |
| APPRAISE-2 (5) | ACS |  | Apixaban | Placebo | 241 days |  | CV death, MI, or ischemic stroke |
| ARISTOTLE (6) | AF |  | Apixaban | Warfarin | 1.8 years |  | Ischemic or hemorrhagic stroke or systemic embolism. |
| ASSENT-3 (7) | AMI |  | Enoxaparin | UFH | 30 days |  | 30-day mortality, in-hospital reinfarction or in-hospital refractory ischaemia |
| ASSENT-3 (7) | AMI |  | Abciximab | UFH | 30 days |  | 30-day mortality, in-hospital reinfarction or in-hospital refractory ischaemia |
| ATHENA (8) | AF |  | Dronedarone | Placebo | 21 months |  | First hospitalization due to CV events or death. |
| ATLAS ACS 2-TIMI 51 (9) | ACS |  | Rivaroxaban | Placebo | 31 months |  | CV death, MI or stroke. |
| ATMOSPHERE (10) | HFrEF |  | Combination (aliskiren / enalapril) | Enalapril | 36.6 months |  | CV death or hospitalization for HF. |
| ATMOSPHERE (10) | HFrEF |  | Aliskiren | Enalapril | 36.6 months |  | CV death or hospitalization for HF. |
| BEAUTIFUL (11) | HFrEF |  | Ivabradine | Placebo | 2.8 years |  | CV death or nonfatal MI. |
| CAROLINA (12) | DM2 |  | Linagliptin | Glimepiride | 18 months |  | CV death, nonfatal MI or nonfatal stroke. |
| CLARITY-TIMI 28 (13) | STEMI |  | Clopidogrel | Placebo | 30 days |  | Occluded infarct-related artery or death or recurrent MI before angiography. |
| CLEAR Outcomes (14) | Statin intolerant + HRCVD |  | Bempedoic Acid | Placebo | 40.6 months |  | Major adverse CV events: CV death, nonfatal MI, nonfatal stroke or coronary revascularization. |
| COMMANDER (15) | HFrEF |  | Rivaroxaban | Placebo | 21.1 months |  | Death from any cause, MI or stroke. |
| COMPASS (16) | ASCVD |  | Rivaroxaban+aspirin | Aspirin | 23 months |  | CV death, stroke or MI. |
| CORONA (17) | HFrEF |  | Rosuvastatin | Placebo | 32.8 months |  | CV death, nonfatal MI or nonfatal stroke. |
| dal-GenE (18) | ACS |  | Dalcetrapib | Placebo | 39.9 months |  | CV death, resuscitated cardiac arrest, nonfatal MI or nonfatal stroke. |
| Dal-OUTCOMES (19) | ACS |  | Dalcetrapib | Placebo | 31 months |  | Death from CHD, nonfatal MI, ischemic stroke, UA or cardiac arrest with resuscitation. |
| DAPA-HF (20) | HFrEF |  | Dapagliflozin | Placebo | 18.2 months |  | Worsening HF or CV death. |
| DECLARE-TIMI 58 (21) | DM2 |  | Dapagliflozin | Placebo | 4.2 years |  | CV death, MI or ischemic stroke. |
| DELIVER (22) | HFmrEF + HFpEF |  | Dapagliflozin | Placebo | 2.3 years |  | Worsening HF or CV death. |
| ELIXA (23) | DM2+MI/UA |  | Lixisenatide | Placebo | 25 months |  | CV death, MI, stroke or hospitalization for UA. |
| EMPACT-MI (24) | AMI |  | Empagliflozin | Placebo | 17.9 months |  | Death from any cause or hospitalization for HF. |
| EMPA-REG OUTCOME (25) | DM2 |  | Empagliflozin | Placebo | 3.1 years |  | CV death, nonfatal MI or nonfatal stroke. |
| EMPEROR-Preserved (26) | HFpEF |  | Empagliflozin | Placebo | 26.2 months |  | CV death or hospitalization for HF. |
| EMPEROR-Reduced (27) | HFrEF |  | Empagliflozin | Placebo | 16 months |  | CV death or hospitalization for worsening HF. |
|  |  |  |  |  |  |  |  |
| EMPHASIS-HF (28) | HFrEF |  | Eplerenone | Placebo | 21 months |  | CV death or hospitalization for HF. |
| EXSCEL (29) | DM2 |  | Exenatide | Placebo | 3.2 years |  | CV death, nonfatal MI or nonfatal stroke. |
| FOURIER (30) | ASCVD |  | Evolocumab | Placebo | 2.2 years |  | CV death, MI, stroke, hospitalization for UA or coronary revascularization. |
| GALACTIC-HF (31) | HFrEF |  | Omecamtiv mecarbil | Placebo | 21.8 months |  | CV death or first HF event. |
| Harmony Outcomes (32) | DM2 + CVD |  | Albiglutide | Placebo | 1.6 years |  | CV death, MI or stroke. |
| Improve-IT (33) | ACS |  | Simvastatin + ezetimibe | Simvastatin+ placebo | 6 years |  | CV death, nonfatal MI, UA requiring rehospitalization, coronary revascularization or nonfatal stroke. |
| LATITUDE-TIMI 60 (34) | AMI |  | Losmapimod | Placebo | 24 weeks |  | CV death, MI or severe recurrent ischemia requiring urgent coronary revascularization. |
| LoDoCo2 (35) | CCD |  | Colchicine | Placebo | 28.6 months |  | CV death, spontaneous MI, ischemic stroke or ischemia-driven coronary revascularization. |
| ODYSSEY OUTCOMES (36) | ACS |  | Alirocumab | Placebo | 2.8 years |  | Death from CHD, nonfatal MI, fatal or nonfatal ischemic stroke or UA requiring hospitalization. |
| ORIGIN (37) | DM2 + HRCVD |  | Insulin Glargine | Standard Care | 6.2 years |  | CV death, nonfatal MI or nonfatal stroke. |
| PACIFIC-AMI (38) | AMI |  | Oral asundexian | Placebo | 368 days |  | CV death, MI, stroke or stent thrombosis. |
| PADIT (39) | Cardiac rhythm devices |  | Incremental | Conventional antibiotics | 1 year |  | Hospitalization for device infection |
| PARADIGM-HF (40) | HFrEF |  | LCZ696 | Enalapril | 27 months |  | CV death or hospitalization for HF. |
| PARADISE-MI (41) | AMI |  | Sacubitril-Valsartan (ARNI) | Ramipril | 22 months |  | CV death or incident HF. |
| PARAGON-HF (42) | HFpEF |  | Sacubitril-Valsartan (ARNI) | Valsartan | 35 months |  | CV death and total hospitalizations for HF. |
| PEGASUS-TIMI 54 (43) | AMI |  | Ticagrelor 90 mg | Ticagrelor 60 mg | 33 months |  | CV death, MI or stroke. |
| PEGASUS-TIMI 54 (43) | AMI |  | Ticagrelor 60 mg | Placebo | 33 months |  | CV death, MI or stroke. |
| PLATO (44) | ACS |  | Ticagrelor | Clopidogrel | 12 months |  | Death from vascular causes, MI or stroke. |
| PROMINENT (45) | DM2 + HRCVD |  | Pemafibrate | Placebo | 3.4 years |  | CV death, nonfatal MI, ischemic stroke or coronary revascularization. |
| RACE (46) | AF |  | Rate-control | Rhythm-control | 3.5 years |  | Overall mortality. |
| RED-HF (47) | HFrEF |  | Darbepoetin Alfa | Placebo | 28 months |  | Death from any cause or hospitalization for worsening HF. |
| REDUCE-IT (48) | HRCVD + high triglyceride |  | Icosapent Ethyl | Placebo | 4.9 years |  | CV death, nonfatal MI, nonfatal stroke, coronary revascularization or UA. |
| RELAX-AHF-2 (49) | AHF |  | Serelaxin | Placebo | 167 days |  | CV death through 180 days of follow-up. |
| ROCKET-AF (50) | AF |  | Rivaroxaban | Warfarin | 707 days |  | Stroke or systemic embolism. |
| SAVOR-TIMI 53 (51) | DM2 + HRCVD |  | Saxagliptin | Placebo | 2.1 years |  | CV death, MI or ischemic stroke. |
| SHIFT (52) | HFrEF |  | Ivabradine | Placebo | 22.9 months |  | CV death or hospital admission for worsening HF. |
| SIGNIFY (53) | CCD |  | Ivabradine | Placebo | 27.8 months |  | CV death or nonfatal MI. |
| SOLID-TIMI 52 (54) | ACS |  | Darapladib | Placebo | 2.5 years |  | Death from CHD, MI or urgent coronary revascularization for myocardial ischemia. |
| SOLOIST-WHF (55) | HF + DM2 |  | Sotagliflozin | Placebo | 9.0 months |  | CV deaths and hospitalizations and urgent visits for HF. |
| Stability LPL II (56) | CCD |  | Darapladib | Placebo | 3.7 years |  | CV death, MI or stroke. |
| STRENGTH (57) | HRCVD |  | Omega-3 CA | Corn oil | 42.0 months |  | CV death, nonfatal MI, nonfatal stroke, coronary revascularization or UA requiring hospitalization. |
| TECOS (58) | History of CVD + DM2 |  | Sitagliptin | Placebo | 3.0 years |  | CV death, nonfatal MI, nonfatal stroke or hospitalization for UA. |
| THEMIS (59) | CCD + DM2 |  | Ticagrelor | Placebo | 39.9 months |  | CV death, MI or stroke. |
| TRA 2P-TIMI 50 (60) | HRCVD |  | Vorapaxar | Placebo | 30 months |  | CV death, MI or stroke |
| TRACER (61) | ACS |  | Vorapaxar | Placebo | 502 days |  | CV death, MI, stroke, recurrent ischemia with rehospitalization or urgent coronary revascularization. |
| TRILOGY ACS (62) | ACS |  | Prasugrel | Clopidogrel | 17 months |  | CV death, MI or stroke. |
| TRUE-AHF (63) | AHF |  | Ularitide | Placebo | 15 months |  | CV death |
| VICTORIA (64) | HFrEF |  | Vericiguat | Placebo | 10.8 months |  | CV death or first hospitalization for HF. |
| VISTA-16 (65) | ACS |  | Varespladib | Placebo | 16 weeks |  | CV death, nonfatal MI, nonfatal stroke or UA with evidence of ischemia requiring hospitalization. |
| SELECT (66) | Previous AMI / stroke + obesity |  | Semaglutide | Placebo | 34.2 months |  | CV death, nonfatal MI or nonfatal stroke. |

Abbreviations used: HRCVD = high risk cardiovascular disease; CV = cardiovascular, MI = myocardial infarction; UA = unstable angina; SAP = stable angina pectoris, AMI = acute myocardial infarction; AHF = acute heart failure; HF = heart failure; ACS = acute coronary syndrome; AF = atrial fibrillation; HFrEF = heart failure with reduced ejection fraction; CAD = coronary artery disease; ASCVD = atherosclerotic cardiovascular disease; DM2 = diabetes mellitus type 2; CHD = coronary heart disease; HFmrEF = heart failure with mid-range ejection fraction; HFpEF = heart failure with preserved ejection fraction; UAP = unstable angina pectoris; CVD = cardiovascular disease; CCD = chronic coronary disease.

**References:**

1. Lincoff AM, Nicholls SJ, Riesmeyer JS, Barter PJ, Brewer HB, Fox KAA, et al. Evacetrapib and Cardiovascular Outcomes in High-Risk Vascular Disease. N Engl J Med. 2017;376(20):1933-42.

2. Poole-Wilson PA, Lubsen J, Kirwan BA, van Dalen FJ, Wagener G, Danchin N, et al. Effect of long-acting nifedipine on mortality and cardiovascular morbidity in patients with stable angina requiring treatment (ACTION trial): randomised controlled trial. Lancet. 2004;364(9437):849-57.

3. Gibson CM, Duffy D, Korjian S, Bahit MC, Chi G, Alexander JH, et al. Apolipoprotein A1 Infusions and Cardiovascular Outcomes after Acute Myocardial Infarction. N Engl J Med. 2024;390(17):1560-71.

4. Ponikowski P, Kirwan BA, Anker SD, McDonagh T, Dorobantu M, Drozdz J, et al. Ferric carboxymaltose for iron deficiency at discharge after acute heart failure: a multicentre, double-blind, randomised, controlled trial. Lancet. 2020;396(10266):1895-904.

5. Alexander JH, Lopes RD, James S, Kilaru R, He Y, Mohan P, et al. Apixaban with antiplatelet therapy after acute coronary syndrome. N Engl J Med. 2011;365(8):699-708.

6. Granger CB, Alexander JH, McMurray JJ, Lopes RD, Hylek EM, Hanna M, et al. Apixaban versus warfarin in patients with atrial fibrillation. N Engl J Med. 2011;365(11):981-92.

7. Assessment of the S, Efficacy of a New Thrombolytic Regimen I. Efficacy and safety of tenecteplase in combination with enoxaparin, abciximab, or unfractionated heparin: the ASSENT-3 randomised trial in acute myocardial infarction. Lancet. 2001;358(9282):605-13.

8. Hohnloser SH, Crijns HJ, van Eickels M, Gaudin C, Page RL, Torp-Pedersen C, et al. Effect of dronedarone on cardiovascular events in atrial fibrillation. N Engl J Med. 2009;360(7):668-78.

9. Mega JL, Braunwald E, Wiviott SD, Bassand JP, Bhatt DL, Bode C, et al. Rivaroxaban in patients with a recent acute coronary syndrome. N Engl J Med. 2012;366(1):9-19.

10. McMurray JJ, Krum H, Abraham WT, Dickstein K, Køber LV, Desai AS, et al. Aliskiren, Enalapril, or Aliskiren and Enalapril in Heart Failure. N Engl J Med. 2016;374(16):1521-32.

11. Fox K, Ford I, Steg PG, Tendera M, Ferrari R, Investigators B. Ivabradine for patients with stable coronary artery disease and left-ventricular systolic dysfunction (BEAUTIFUL): a randomised, double-blind, placebo-controlled trial. Lancet. 2008;372(9641):807-16.

12. Rosenstock J, Kahn SE, Johansen OE, Zinman B, Espeland MA, Woerle HJ, et al. Effect of Linagliptin vs Glimepiride on Major Adverse Cardiovascular Outcomes in Patients With Type 2 Diabetes: The CAROLINA Randomized Clinical Trial. Jama. 2019;322(12):1155-66.

13. Sabatine MS, Cannon CP, Gibson CM, López-Sendón JL, Montalescot G, Theroux P, et al. Addition of clopidogrel to aspirin and fibrinolytic therapy for myocardial infarction with ST-segment elevation. N Engl J Med. 2005;352(12):1179-89.

14. Nissen SE, Lincoff AM, Brennan D, Ray KK, Mason D, Kastelein JJP, et al. Bempedoic Acid and Cardiovascular Outcomes in Statin-Intolerant Patients. N Engl J Med. 2023;388(15):1353-64.

15. Zannad F, Anker SD, Byra WM, Cleland JGF, Fu M, Gheorghiade M, et al. Rivaroxaban in Patients with Heart Failure, Sinus Rhythm, and Coronary Disease. N Engl J Med. 2018;379(14):1332-42.

16. Eikelboom JW, Connolly SJ, Bosch J, Dagenais GR, Hart RG, Shestakovska O, et al. Rivaroxaban with or without Aspirin in Stable Cardiovascular Disease. New England Journal of Medicine. 2017;377(14):1319-30.

17. Kjekshus J, Apetrei E, Barrios V, Böhm M, Cleland JG, Cornel JH, et al. Rosuvastatin in older patients with systolic heart failure. N Engl J Med. 2007;357(22):2248-61.

18. Tardif JC, Pfeffer MA, Kouz S, Koenig W, Maggioni AP, McMurray JJV, et al. Pharmacogenetics-guided dalcetrapib therapy after an acute coronary syndrome: the dal-GenE trial. Eur Heart J. 2022;43(39):3947-56.

19. Schwartz GG, Olsson AG, Abt M, Ballantyne CM, Barter PJ, Brumm J, et al. Effects of dalcetrapib in patients with a recent acute coronary syndrome. N Engl J Med. 2012;367(22):2089-99.

20. McMurray JJV, Solomon SD, Inzucchi SE, Køber L, Kosiborod MN, Martinez FA, et al. Dapagliflozin in Patients with Heart Failure and Reduced Ejection Fraction. N Engl J Med. 2019;381(21):1995-2008.

21. Wiviott SD, Raz I, Bonaca MP, Mosenzon O, Kato ET, Cahn A, et al. Dapagliflozin and Cardiovascular Outcomes in Type 2 Diabetes. N Engl J Med. 2019;380(4):347-57.

22. Solomon SD, McMurray JJV, Claggett B, de Boer RA, DeMets D, Hernandez AF, et al. Dapagliflozin in Heart Failure with Mildly Reduced or Preserved Ejection Fraction. N Engl J Med. 2022;387(12):1089-98.

23. Pfeffer MA, Claggett B, Diaz R, Dickstein K, Gerstein HC, Køber LV, et al. Lixisenatide in Patients with Type 2 Diabetes and Acute Coronary Syndrome. N Engl J Med. 2015;373(23):2247-57.

24. Butler J, Jones WS, Udell JA, Anker SD, Petrie MC, Harrington J, et al. Empagliflozin after Acute Myocardial Infarction. N Engl J Med. 2024;390(16):1455-66.

25. Zinman B, Wanner C, Lachin JM, Fitchett D, Bluhmki E, Hantel S, et al. Empagliflozin, Cardiovascular Outcomes, and Mortality in Type 2 Diabetes. N Engl J Med. 2015;373(22):2117-28.

26. Anker SD, Butler J, Filippatos G, Ferreira JP, Bocchi E, Böhm M, et al. Empagliflozin in Heart Failure with a Preserved Ejection Fraction. N Engl J Med. 2021;385(16):1451-61.

27. Packer M, Anker SD, Butler J, Filippatos G, Pocock SJ, Carson P, et al. Cardiovascular and Renal Outcomes with Empagliflozin in Heart Failure. N Engl J Med. 2020;383(15):1413-24.

28. Zannad F, McMurray JJ, Krum H, van Veldhuisen DJ, Swedberg K, Shi H, et al. Eplerenone in patients with systolic heart failure and mild symptoms. N Engl J Med. 2011;364(1):11-21.

29. Holman RR, Bethel MA, Mentz RJ, Thompson VP, Lokhnygina Y, Buse JB, et al. Effects of Once-Weekly Exenatide on Cardiovascular Outcomes in Type 2 Diabetes. N Engl J Med. 2017;377(13):1228-39.

30. Sabatine MS, Giugliano RP, Keech AC, Honarpour N, Wiviott SD, Murphy SA, et al. Evolocumab and Clinical Outcomes in Patients with Cardiovascular Disease. N Engl J Med. 2017;376(18):1713-22.

31. Teerlink JR, Diaz R, Felker GM, McMurray JJV, Metra M, Solomon SD, et al. Cardiac Myosin Activation with Omecamtiv Mecarbil in Systolic Heart Failure. N Engl J Med. 2021;384(2):105-16.

32. Hernandez AF, Green JB, Janmohamed S, D'Agostino RB, Sr., Granger CB, Jones NP, et al. Albiglutide and cardiovascular outcomes in patients with type 2 diabetes and cardiovascular disease (Harmony Outcomes): a double-blind, randomised placebo-controlled trial. Lancet. 2018;392(10157):1519-29.

33. Cannon CP, Blazing MA, Giugliano RP, McCagg A, White JA, Theroux P, et al. Ezetimibe Added to Statin Therapy after Acute Coronary Syndromes. N Engl J Med. 2015;372(25):2387-97.

34. O'Donoghue ML, Glaser R, Cavender MA, Aylward PE, Bonaca MP, Budaj A, et al. Effect of Losmapimod on Cardiovascular Outcomes in Patients Hospitalized With Acute Myocardial Infarction: A Randomized Clinical Trial. Jama. 2016;315(15):1591-9.

35. Nidorf SM, Fiolet ATL, Mosterd A, Eikelboom JW, Schut A, Opstal TSJ, et al. Colchicine in Patients with Chronic Coronary Disease. N Engl J Med. 2020;383(19):1838-47.

36. Schwartz GG, Steg PG, Szarek M, Bhatt DL, Bittner VA, Diaz R, et al. Alirocumab and Cardiovascular Outcomes after Acute Coronary Syndrome. N Engl J Med. 2018;379(22):2097-107.

37. Investigators OT, Gerstein HC, Bosch J, Dagenais GR, Díaz R, Jung H, et al. Basal insulin and cardiovascular and other outcomes in dysglycemia. N Engl J Med. 2012;367(4):319-28.

38. Rao SV, Kirsch B, Bhatt DL, Budaj A, Coppolecchia R, Eikelboom J, et al. A Multicenter, Phase 2, Randomized, Placebo-Controlled, Double-Blind, Parallel-Group, Dose-Finding Trial of the Oral Factor XIa Inhibitor Asundexian to Prevent Adverse Cardiovascular Outcomes After Acute Myocardial Infarction. Circulation. 2022;146(16):1196-206.

39. Krahn AD, Longtin Y, Philippon F, Birnie DH, Manlucu J, Angaran P, et al. Prevention of Arrhythmia Device Infection Trial: The PADIT Trial. J Am Coll Cardiol. 2018;72(24):3098-109.

40. McMurray JJ, Packer M, Desai AS, Gong J, Lefkowitz MP, Rizkala AR, et al. Angiotensin-neprilysin inhibition versus enalapril in heart failure. N Engl J Med. 2014;371(11):993-1004.

41. Pfeffer MA, Claggett B, Lewis EF, Granger CB, Køber L, Maggioni AP, et al. Angiotensin Receptor-Neprilysin Inhibition in Acute Myocardial Infarction. N Engl J Med. 2021;385(20):1845-55.

42. Solomon SD, McMurray JJV, Anand IS, Ge J, Lam CSP, Maggioni AP, et al. Angiotensin-Neprilysin Inhibition in Heart Failure with Preserved Ejection Fraction. N Engl J Med. 2019;381(17):1609-20.

43. Bonaca MP, Bhatt DL, Cohen M, Steg PG, Storey RF, Jensen EC, et al. Long-term use of ticagrelor in patients with prior myocardial infarction. N Engl J Med. 2015;372(19):1791-800.

44. Wallentin L, Becker RC, Budaj A, Cannon CP, Emanuelsson H, Held C, et al. Ticagrelor versus clopidogrel in patients with acute coronary syndromes. N Engl J Med. 2009;361(11):1045-57.

45. Das Pradhan A, Glynn RJ, Fruchart JC, MacFadyen JG, Zaharris ES, Everett BM, et al. Triglyceride Lowering with Pemafibrate to Reduce Cardiovascular Risk. N Engl J Med. 2022;387(21):1923-34.

46. Van Gelder IC, Hagens VE, Bosker HA, Kingma JH, Kamp O, Kingma T, et al. A Comparison of Rate Control and Rhythm Control in Patients with Recurrent Persistent Atrial Fibrillation. New England Journal of Medicine. 2002;347(23):1834-40.

47. Swedberg K, Young JB, Anand IS, Cheng S, Desai AS, Diaz R, et al. Treatment of anemia with darbepoetin alfa in systolic heart failure. N Engl J Med. 2013;368(13):1210-9.

48. Bhatt DL, Steg PG, Miller M, Brinton EA, Jacobson TA, Ketchum SB, et al. Cardiovascular Risk Reduction with Icosapent Ethyl for Hypertriglyceridemia. N Engl J Med. 2019;380(1):11-22.

49. Metra M, Teerlink JR, Cotter G, Davison BA, Felker GM, Filippatos G, et al. Effects of Serelaxin in Patients with Acute Heart Failure. N Engl J Med. 2019;381(8):716-26.

50. Patel MR, Mahaffey KW, Garg J, Pan G, Singer DE, Hacke W, et al. Rivaroxaban versus warfarin in nonvalvular atrial fibrillation. N Engl J Med. 2011;365(10):883-91.

51. Scirica BM, Bhatt DL, Braunwald E, Steg PG, Davidson J, Hirshberg B, et al. Saxagliptin and cardiovascular outcomes in patients with type 2 diabetes mellitus. N Engl J Med. 2013;369(14):1317-26.

52. Swedberg K, Komajda M, Böhm M, Borer JS, Ford I, Dubost-Brama A, et al. Ivabradine and outcomes in chronic heart failure (SHIFT): a randomised placebo-controlled study. Lancet. 2010;376(9744):875-85.

53. Fox K, Ford I, Steg PG, Tardif JC, Tendera M, Ferrari R, Investigators S. Ivabradine in stable coronary artery disease without clinical heart failure. N Engl J Med. 2014;371(12):1091-9.

54. O'Donoghue ML, Braunwald E, White HD, Lukas MA, Tarka E, Steg PG, et al. Effect of darapladib on major coronary events after an acute coronary syndrome: the SOLID-TIMI 52 randomized clinical trial. Jama. 2014;312(10):1006-15.

55. Bhatt DL, Szarek M, Steg PG, Cannon CP, Leiter LA, McGuire DK, et al. Sotagliflozin in Patients with Diabetes and Recent Worsening Heart Failure. N Engl J Med. 2021;384(2):117-28.

56. Investigators S, White HD, Held C, Stewart R, Tarka E, Brown R, et al. Darapladib for preventing ischemic events in stable coronary heart disease. N Engl J Med. 2014;370(18):1702-11.

57. Nicholls SJ, Lincoff AM, Garcia M, Bash D, Ballantyne CM, Barter PJ, et al. Effect of High-Dose Omega-3 Fatty Acids vs Corn Oil on Major Adverse Cardiovascular Events in Patients at High Cardiovascular Risk: The STRENGTH Randomized Clinical Trial. Jama. 2020;324(22):2268-80.

58. Green JB, Bethel MA, Armstrong PW, Buse JB, Engel SS, Garg J, et al. Effect of Sitagliptin on Cardiovascular Outcomes in Type 2 Diabetes. N Engl J Med. 2015;373(3):232-42.

59. Steg PG, Bhatt DL, Simon T, Fox K, Mehta SR, Harrington RA, et al. Ticagrelor in Patients with Stable Coronary Disease and Diabetes. N Engl J Med. 2019;381(14):1309-20.

60. Morrow DA, Braunwald E, Bonaca MP, Ameriso SF, Dalby AJ, Fish MP, et al. Vorapaxar in the secondary prevention of atherothrombotic events. N Engl J Med. 2012;366(15):1404-13.

61. Tricoci P, Huang Z, Held C, Moliterno DJ, Armstrong PW, Van de Werf F, et al. Thrombin-receptor antagonist vorapaxar in acute coronary syndromes. N Engl J Med. 2012;366(1):20-33.

62. Roe MT, Armstrong PW, Fox KA, White HD, Prabhakaran D, Goodman SG, et al. Prasugrel versus clopidogrel for acute coronary syndromes without revascularization. N Engl J Med. 2012;367(14):1297-309.

63. Packer M, O'Connor C, McMurray JJV, Wittes J, Abraham WT, Anker SD, et al. Effect of Ularitide on Cardiovascular Mortality in Acute Heart Failure. N Engl J Med. 2017;376(20):1956-64.

64. Armstrong PW, Pieske B, Anstrom KJ, Ezekowitz J, Hernandez AF, Butler J, et al. Vericiguat in Patients with Heart Failure and Reduced Ejection Fraction. N Engl J Med. 2020;382(20):1883-93.

65. Nicholls SJ, Kastelein JJ, Schwartz GG, Bash D, Rosenson RS, Cavender MA, et al. Varespladib and cardiovascular events in patients with an acute coronary syndrome: the VISTA-16 randomized clinical trial. Jama. 2014;311(3):252-62.

66. Lincoff AM, Brown-Frandsen K, Colhoun HM, Deanfield J, Emerson SS, Esbjerg S, et al. Semaglutide and Cardiovascular Outcomes in Obesity without Diabetes. N Engl J Med. 2023;389(24):2221-32.
