# Supplementary material for: A comprehensive analysis of female participation in cardiovascular trials involving the WCN investigator network
Source: Neth Heart J. 2025 Nov 12;33(12):404–11. doi: 10.1007/s12471-025-01999-4 (PMC12638513; doi:10.1007/s12471-025-01999-4)
Supplement: Supplementary file 4 — Table S3 B. Sex-specific REM of included event-driven studies. [file 12471_2025_1999_MOESM4_ESM.jpg]

| **Trial** |  | **Males** | | | | |  | **Females** | | | | |  | **Relative efficacy measure (REM)** | | | | |
| --- | --- | --- | --- | --- | --- | --- | --- | --- | --- | --- | --- | --- | --- | --- | --- | --- | --- | --- |
|  |  | **Experimental** | |  | **Control** | |  | **Experimental** | |  | **Control** | |  | **Measure** |  | **Males** |  | **Females** |
|  |  | **N** | **EP (%)** |  | **N** | **EP (%)** |  | **N** | **EP (%)** |  | **N** | **EP (%)** |  |  |  | **Point estimate (95% CI)** |  | **Point estimate (95% CI)** |
| ACCELERATE (1) |  | 4648 | 13.0 |  | 4660 | 12.5 |  | 1390 | 12.7 |  | 1394 | 13.8 |  | HR |  | 1.04 (0.93-0.97) |  | 0.91 (0.74-1.12) |
| ACTION (2) |  | 3041 | 21.0 |  | 3043 | 22.4 |  | 784 | 21.2 |  | 797 | 18.4 |  | HR |  | 0.93 (0.83-1.03) |  | 1.16 (0.93-1.45) |
| AEGIS-II (3) |  | 6786 | 4.6 |  | 6721 | 5.0 |  | 2326 | 5.4 |  | 2386 | 5.8 |  | HR |  | 0.93 (0.80-1.09) |  | 0.91 (0.71-1.16) |
| AFFIRM-AHF (4) |  | 314 | 54.8 |  | 300 | 79.3 |  | 244 | 49.6 |  | 250 | 53.6 |  | RR |  | 0.64 (0.46-0.89) |  | 1.05 (0.72-1.53) |
| APPRAISE-2 (5) |  | 2496 |  |  | 2518 |  |  | 1209 |  |  | 1169 |  |  | HR |  | 0.88 (0.71-1.08) |  | 1.08 (0.83-1.42) |
| ARISTOTLE (6) |  | 5886 | 2.2 |  | 5899 | 2.7 |  | 3234 | 2.5 |  | 3182 | 3.3 |  | HR |  | 0.82 (0.65-1.04) |  | 0.74 (0.56-1.00) |
| ASSENT-3 (7) |  | 1574 | 11.9 |  | 1558 | 16.2 |  | 463 | 20.1 |  | 478 | 19.9 |  | HR |  | 0.70 (0.58-0.84) |  | 0.87 (0.65-1.17) |
| ASSENT-3 (7) |  | 1522 | 12.3 |  | 1558 | 16.2 |  | 494 | 20.2 |  | 478 | 19.9 |  | HR |  | 0.66 (0.55-0.80) |  | 0.85 (0.64-1.14) |
| ATHENA (8) |  | 1170 |  |  | 1289 |  |  | 1131 |  |  | 1038 |  |  | HR |  | 0.74 (0.64-0.85) |  | 0.77 (0.60-0.99) |
| ATLAS ACS 2-TIMI 51 (9) |  | 7627 | 6.1 |  | 3831 | 7.0 |  | 2602 | 6.2 |  | 1282 | 8.3 |  | HR |  | 0.87 (0.75-1.01) |  | 0.77 (0.60-0.99) |
| ATMOSPHERE (10) |  | 1846 | 34.3 |  | 1837 | 36.4 |  | 494 | 27.7 |  | 499 | 28.1 |  | HR |  | 0.92 (0.82-1.02) |  | 0.99 (0.78-1.25) |
| ATMOSPHERE (10) |  | 1808 | 35.0 |  | 1837 | 36.4 |  | 532 | 29.7 |  | 499 | 28.1 |  | HR |  | 0.95 (0.85-1.06) |  | 1.21 (0.96-1.52) |
| BEAUTIFUL (11) |  | 4540 | 15.2 |  | 4507 | 15.5 |  | 939 | 16.2 |  | 931 | 14.2 |  | HR |  | 0.98 (0.88-1.08) |  | 1.14 (0.90-1.44) |
| CAROLINA (12) |  | 1838 | 13.6 |  | 1781 | 14.8 |  | 1185 | 8.9 |  | 1229 | 8.1 |  | HR |  | 0.92 (0.77-1.09) |  | 1.11 (0.84-1.46) |
| CLARITY-TIMI 28 (13) |  | 1400 | 14.5 |  | 1403 | 20.8 |  | 352 | 16.9 |  | 336 | 24.7 |  | OR |  | 0.65 (0.52-0.80) |  | 0.63 (0.43-0.92) |
| CLEAR Outcomes (14) |  | 3631 | 14.8 |  | 3599 | 16.6 |  | 3361 | 8.4 |  | 3379 | 9.7 |  | HR |  | 0.87 (0.77-0.98) |  | 0.86 (0.73-1.01) |
| COMMANDER (15) |  | 1956 | 24.9 |  | 1916 | 27.0 |  | 551 | 25.2 |  | 599 | 23.5 |  | HR |  | 0.92 (0.81-1.04) |  | 1.05 (0.83-1.33) |
| COMPASS (16) |  | 7093 | 4.2 |  | 7137 | 5.5 |  | 2059 | 3.8 |  | 1989 | 5.2 |  | HR |  | 0.76 (0.66-0.89) |  | 0.72 (0.54-0.97) |
| CORONA (17) |  | 1921 | 28.8 |  | 1910 | 30.2 |  | 593 | 23.3 |  | 587 | 26.4 |  | HR |  | 0.94 (0.84-1.05) |  | 0.86 (0.68-1.09) |
| dal-GenE (18) |  | 2348 | 9.6 |  | 2404 | 10.5 |  | 723 | 9.3 |  | 672 | 11.2 |  | HR |  | 0.90 (0.75-1.08) |  | 0.82 (0.59-1.13) |
| Dal-OUTCOMES (19) |  | 6365 |  |  | 6436 |  |  | 1573 |  |  | 1497 |  |  | HR |  | 1.07 (0.95-1.12) |  | 0.92 (0.72-1.16) |
| DAPA-HF (20) |  | 1809 | 17.0 |  | 1826 | 22.2 |  | 564 | 14.0 |  | 545 | 17.6 |  | HR |  | 0.73 (0.63-0.85) |  | 0.79 (0.59-1.06) |
| DECLARE-TIMI 58 (21) |  | 5411 | 10.2 |  | 5327 | 10.9 |  | 3171 | 6.4 |  | 3251 | 6.9 |  | HR |  | 0.94 (0.83-1.06) |  | 0.94 (0.79-1.13) |
| DELIVER (22) |  | 1767 | 17.9 |  | 1749 | 21.0 |  | 1364 | 14.3 |  | 1383 | 17.6 |  | HR |  | 0.82 (0.71-0.96) |  | 0.81 (0.67-0.97) |
| ELIXA (23) |  | 2031 |  |  | 1994 |  |  | 1003 |  |  | 1040 |  |  | HR |  | 1.06 (0.89-1.25) |  | 0.91 (0.71-1.18) |
| EMPACT-MI (24) |  | 2448 | 7.2 |  | 2449 | 8.9 |  | 812 | 11.2 |  | 813 | 9.7 |  | HR |  | 0.81 (0.66-0.99) |  | 1.14 (0.85-1.55) |
| EMPA-REG OUTCOME (25) |  | 3336 | 11.0 |  | 1680 | 12.6 |  | 1351 | 9.1 |  | 653 | 10.7 |  | HR |  | 0.87 (0.73-1.02) |  | 0.83 (0.62-1.11) |
| EMPEROR-Preserved (26) |  | 1659 | 15.3 |  | 1653 | 18.0 |  | 1338 | 12.1 |  | 1338 | 16.0 |  | HR |  | 0.81 (0.69-0.96) |  | 0.75 (0.61-0.92) |
| EMPEROR-Reduced (27) |  | 1426 | 20.6 |  | 1411 | 25.0 |  | 437 | 15.3 |  | 456 | 23.9 |  | HR |  | 0.80 (0.68-0.93) |  | 0.59 (0.44-0.80) |
| EMPHASIS-HF (28) |  | 1055 |  |  | 1072 |  |  | 309 |  |  | 301 |  |  | HR |  | 0.72 (0.62-0.85) |  | 0.61 (0.43-0.85) |
| EXSCEL (29) |  | 4562 | 13.1 |  | 4587 | 13.8 |  | 2794 | 8.6 |  | 2809 | 9.6 |  | HR |  | 0.94 (0.84-1.05) |  | 0.86 (0.73-1.03) |
| FOURIER (30) |  | 10397 |  |  | 10398 |  |  | 3387 |  |  | 3382 |  |  | HR |  | 0.86 (0.80-0.94) |  | 0.81 (0.69-0.95) |
| GALACTIC-HF (31) |  | 3245 |  |  | 3238 |  |  | 875 |  |  | 874 |  |  | HR |  | 0.92 (0.85-0.99) |  | 0.95 (0.81-1.12) |
| Harmony Outcomes (32) |  | 3304 | 7.8 |  | 3265 | 9.4 |  | 1427 | 5.5 |  | 1467 | 8.2 |  | HR |  | 0.82 (0.69-0.97) |  | 0.67 (0.50-0.89) |
| Improve-IT (33) |  | 6842 |  |  | 6886 |  |  | 2225 |  |  | 2191 |  |  | HR |  | 0.95 (0.90-1.01) |  | 0.89 (0.79-0.99) |
| LATITUDE-TIMI 60 (34) |  | 1231 | 7.6 |  | 1226 | 6.5 |  | 500 | 9.0 |  | 532 | 8.1 |  | HR |  | 1.18 (0.87-1.59) |  | 1.13 (0.74-1.71) |
| LoDoCo2 (35) |  | 2305 | 6.9 |  | 2371 | 1 |  | 457 | 6.1 |  | 389 | 7.2 |  | HR |  | 0.68 (0.55-0.83) |  | 0.81 (0.48-1.37) |
| ODYSSEY OUTCOMES (36) |  | 6842 |  |  | 6886 |  |  | 2225 |  |  | 2191 |  |  | HR |  | 0.83 (0.74-0.92) |  | 0.91 (0.77-1.08) |
| ORIGIN (37) |  | 4182 | 18.0 |  | 3969 | 18.2 |  | 2082 | 13.8 |  | 2304 | 12.7 |  | HR |  | 0.98 (0.89-1.09) |  | 1.11 (0.94-1.31) |
| PACIFIC-AMI (38) |  | 920 | 8.3 |  | 311 | 8.7 |  | 280 | 1 |  | 90 | 1 |  | csHR |  | 0.95 (0.65-1.37) |  | 1.07 (0.57-2.01) |
| PADIT (39) |  | 6655 | 0.9 |  | 6267 | 1.1 |  | 3299 | 0.5 |  | 3338 | 0.8 |  | OR |  | 0.79 (0.55-1.13) |  | 0.68 (0.37-1.25) |
| PARADIGM-HF (40) |  | 3308 |  |  | 3259 |  |  | 879 |  |  | 953 |  |  | HR |  | 0.81 (0.72-0.89) |  | 0.77 (0.62-0.94) |
| PARADISE-MI (41) |  | 2167 |  |  | 2131 |  |  | 663 |  |  | 700 |  |  | HR |  | 0.84 (0.71-1.00) |  | 1.10 (0.83-1.45) |
| PARAGON-HF (42) |  | 1166 |  |  | 1151 |  |  | 1241 |  |  | 1238 |  |  | HR |  | 1.03 (0.85-1.25) |  | 0.73 (0.59-0.90) |
| PEGASUS-TIMI 54 (43) |  | 5368 |  |  | 5384 |  |  | 1682 |  |  | 1661 |  |  | HR |  | 0.89 (0.77-1.02) |  | 0.74 (0.57-0.95) |
| PEGASUS-TIMI 54 (43) |  | 5384 |  |  | 5350 |  |  | 1661 |  |  | 1717 |  |  | HR |  | 0.79 (0.69-0.91) |  | 0.98 (0.78-1.24) |
| PLATO (44) |  | 6678 |  |  | 6658 |  |  | 2655 |  |  | 2633 |  |  | HR |  | 0.85 (0.76-0.95) |  | 0.83 (0.71-0.97) |
| PROMINENT (45) |  | 3797 | 12.0 |  | 3809 | 11.8 |  | 1443 | 7.6 |  | 1448 | 8.2 |  | IRR |  | 1.01 (0.89-1.15) |  | 1.08 (0.83-1.40) |
| RACE (46) |  | 161 | 21.1 |  | 170 | 17.1 |  | 95 | 10.5 |  | 96 | 32.3 |  | OR |  | 1.29 (0.75-2.24) |  | 0.25 (0.11-0.55) |
| RED-HF (47) |  | 678 | 58.0 |  | 656 | 55.3 |  | 458 | 4 |  | 486 | 41.6 |  | HR |  | 1.01 (0.87-1.16) |  | 0.96 (0.79-1.18) |
| REDUCE-IT (48) |  | 2927 | 18.8 |  | 2895 | 24.7 |  | 1162 | 13.3 |  | 1195 | 15.6 |  | HR |  | 0.73 (0.65-0.82) |  | 0.82 (0.66-1.01) |
| RELAX-AHF-2 (49) |  | 1978 | 8.5 |  | 1930 | 9.2 |  | 1296 | 9.0 |  | 1341 | 8.4 |  | HR |  | 0.91 (0.74-1.13) |  | 1.08 (0.83-1.40) |
| ROCKET-AF (50) |  | 4279 | 3.3 |  | 4287 | 3.8 |  | 2802 | 4.5 |  | 2803 | 5.1 |  | HR |  | 0.87 (0.70-1.09) |  | 0.89 (0.70-1.12) |
| SAVOR-TIMI 53 (51) |  | 5512 |  |  | 5525 |  |  | 2768 |  |  | 2687 |  |  | HR |  | 1.01 (0.89-1.16) |  | 0.97 (0.78-1.20) |
| SHIFT (52) |  | 2462 | 25.3 |  | 2508 | 28.9 |  | 779 | 21.7 |  | 754 | 28.1 |  | HR |  | 0.84 (0.76-0.94) |  | 0.74 (0.60-0.91) |
| SIGNIFY (53) |  | 6949 | 7.0 |  | 6890 | 6.7 |  | 2601 | 6.5 |  | 2662 | 5.7 |  | HR |  | 1.05 (0.92-1.19) |  | 1.16 (0.93-1.44) |
| SOLID-TIMI 52 (54) |  | 4847 | 13.2 |  | 4853 | 14.1 |  | 1657 | 15.8 |  | 1669 | 13.7 |  | HR |  | 0.94 (0.84-1.05) |  | 1.17 (0.98-1.40) |
| SOLOIST-WHF (55) |  | 410 |  |  | 400 |  |  | 198 |  |  | 214 |  |  | HR |  | 0.62 (0.47-0.82) |  | 0.80 (0.51-1.25) |
| Stability LPL II (56) |  | 6463 | 9.7 |  | 6398 | 10.5 |  | 1461 | 9.9 |  | 1506 | 9.9 |  | HR |  | 0.92 (0.83-1.03) |  | 1.01 (0.80-1.26) |
| STRENGTH (57) |  | 4250 | 13.7 |  | 4260 | 13.6 |  | 2289 | 8.8 |  | 2279 | 9.5 |  | HR |  | 1.01 (0.90-1.13) |  | 0.94 (0.78-1.14) |
| TECOS (58) |  | 5198 |  |  | 5176 |  |  | 2134 |  |  | 2163 |  |  | HR |  | 0.99 (0.88-1.10) |  | 0.95 (0.78-1.15) |
| THEMIS (59) |  | 6576 | 7.7 |  | 6613 | 8.8 |  | 3043 | 7.6 |  | 2988 | 8.0 |  | HR |  | 0.88 (0.78-0.99) |  | 0.96 (0.80-1.15) |
| TRA 2P-TIMI 50 (60) |  | 10071 |  |  | 10052 |  |  | 3154 |  |  | 3172 |  |  | HR |  | 0.85 (0.77-0.94) |  | 0.92 (0.78-1.09) |
| TRACER (61) |  | 4649 |  |  | 4663 |  |  | 1822 |  |  | 1810 |  |  | HR |  | 0.90 (0.81-0.99) |  | 0.99 (0.85-1.17) |
| TRILOGY ACS (62) |  | 2835 |  |  | 2840 |  |  | 1828 |  |  | 1823 |  |  | HR |  | 0.86 (0.72-1.03) |  | 1.02 (0.80-1.29) |
| TRUE-AHF (63) |  | 714 | 22.8 |  | 706 | 22.1 |  | 374 | 19.5 |  | 363 | 19.0 |  | HR |  | 1.01 (0.81-1.26) |  | 1.08 (0.78-1.50) |
| VICTORIA (64) |  | 1921 | 36.6 |  | 1921 | 39.7 |  | 605 | 31.9 |  | 603 | 34.8 |  | HR |  | 0.90 (0.81-1.00) |  | 0.88 (0.73-1.08) |
| VISTA-16 (65) |  | 1881 | 4.6 |  | 1913 | 4.0 |  | 691 | 7.2 |  | 660 | 5.2 |  | HR |  | 1.16 (0.85-1.58) |  | 1.44 (0.93-2.23) |
| SELECT (66) |  | 6355 | 7.0 |  | 6377 | 8.7 |  | 2448 | 5.1 |  | 2424 | 6.1 |  | HR |  | 0.79 (0.70-0.90) |  | 0.84 (0.66-1.07) |

Abbreviations used: HR = hazard ratio; OR = odds ratio; csHR = cause-specific hazard ratio and IRR = incidence rate ratio.

**References:**

1. Lincoff AM, Nicholls SJ, Riesmeyer JS, Barter PJ, Brewer HB, Fox KAA, et al. Evacetrapib and Cardiovascular Outcomes in High-Risk Vascular Disease. N Engl J Med. 2017;376(20):1933-42.

2. Poole-Wilson PA, Lubsen J, Kirwan BA, van Dalen FJ, Wagener G, Danchin N, et al. Effect of long-acting nifedipine on mortality and cardiovascular morbidity in patients with stable angina requiring treatment (ACTION trial): randomised controlled trial. Lancet. 2004;364(9437):849-57.

3. Gibson CM, Duffy D, Korjian S, Bahit MC, Chi G, Alexander JH, et al. Apolipoprotein A1 Infusions and Cardiovascular Outcomes after Acute Myocardial Infarction. N Engl J Med. 2024;390(17):1560-71.

4. Ponikowski P, Kirwan BA, Anker SD, McDonagh T, Dorobantu M, Drozdz J, et al. Ferric carboxymaltose for iron deficiency at discharge after acute heart failure: a multicentre, double-blind, randomised, controlled trial. Lancet. 2020;396(10266):1895-904.

5. Alexander JH, Lopes RD, James S, Kilaru R, He Y, Mohan P, et al. Apixaban with antiplatelet therapy after acute coronary syndrome. N Engl J Med. 2011;365(8):699-708.

6. Granger CB, Alexander JH, McMurray JJ, Lopes RD, Hylek EM, Hanna M, et al. Apixaban versus warfarin in patients with atrial fibrillation. N Engl J Med. 2011;365(11):981-92.

7. Assessment of the S, Efficacy of a New Thrombolytic Regimen I. Efficacy and safety of tenecteplase in combination with enoxaparin, abciximab, or unfractionated heparin: the ASSENT-3 randomised trial in acute myocardial infarction. Lancet. 2001;358(9282):605-13.

8. Hohnloser SH, Crijns HJ, van Eickels M, Gaudin C, Page RL, Torp-Pedersen C, et al. Effect of dronedarone on cardiovascular events in atrial fibrillation. N Engl J Med. 2009;360(7):668-78.

9. Mega JL, Braunwald E, Wiviott SD, Bassand JP, Bhatt DL, Bode C, et al. Rivaroxaban in patients with a recent acute coronary syndrome. N Engl J Med. 2012;366(1):9-19.

10. McMurray JJ, Krum H, Abraham WT, Dickstein K, Køber LV, Desai AS, et al. Aliskiren, Enalapril, or Aliskiren and Enalapril in Heart Failure. N Engl J Med. 2016;374(16):1521-32.

11. Fox K, Ford I, Steg PG, Tendera M, Ferrari R, Investigators B. Ivabradine for patients with stable coronary artery disease and left-ventricular systolic dysfunction (BEAUTIFUL): a randomised, double-blind, placebo-controlled trial. Lancet. 2008;372(9641):807-16.

12. Rosenstock J, Kahn SE, Johansen OE, Zinman B, Espeland MA, Woerle HJ, et al. Effect of Linagliptin vs Glimepiride on Major Adverse Cardiovascular Outcomes in Patients With Type 2 Diabetes: The CAROLINA Randomized Clinical Trial. Jama. 2019;322(12):1155-66.

13. Sabatine MS, Cannon CP, Gibson CM, López-Sendón JL, Montalescot G, Theroux P, et al. Addition of clopidogrel to aspirin and fibrinolytic therapy for myocardial infarction with ST-segment elevation. N Engl J Med. 2005;352(12):1179-89.

14. Nissen SE, Lincoff AM, Brennan D, Ray KK, Mason D, Kastelein JJP, et al. Bempedoic Acid and Cardiovascular Outcomes in Statin-Intolerant Patients. N Engl J Med. 2023;388(15):1353-64.

15. Zannad F, Anker SD, Byra WM, Cleland JGF, Fu M, Gheorghiade M, et al. Rivaroxaban in Patients with Heart Failure, Sinus Rhythm, and Coronary Disease. N Engl J Med. 2018;379(14):1332-42.

16. Eikelboom JW, Connolly SJ, Bosch J, Dagenais GR, Hart RG, Shestakovska O, et al. Rivaroxaban with or without Aspirin in Stable Cardiovascular Disease. New England Journal of Medicine. 2017;377(14):1319-30.

17. Kjekshus J, Apetrei E, Barrios V, Böhm M, Cleland JG, Cornel JH, et al. Rosuvastatin in older patients with systolic heart failure. N Engl J Med. 2007;357(22):2248-61.

18. Tardif JC, Pfeffer MA, Kouz S, Koenig W, Maggioni AP, McMurray JJV, et al. Pharmacogenetics-guided dalcetrapib therapy after an acute coronary syndrome: the dal-GenE trial. Eur Heart J. 2022;43(39):3947-56.

19. Schwartz GG, Olsson AG, Abt M, Ballantyne CM, Barter PJ, Brumm J, et al. Effects of dalcetrapib in patients with a recent acute coronary syndrome. N Engl J Med. 2012;367(22):2089-99.

20. McMurray JJV, Solomon SD, Inzucchi SE, Køber L, Kosiborod MN, Martinez FA, et al. Dapagliflozin in Patients with Heart Failure and Reduced Ejection Fraction. N Engl J Med. 2019;381(21):1995-2008.

21. Wiviott SD, Raz I, Bonaca MP, Mosenzon O, Kato ET, Cahn A, et al. Dapagliflozin and Cardiovascular Outcomes in Type 2 Diabetes. N Engl J Med. 2019;380(4):347-57.

22. Solomon SD, McMurray JJV, Claggett B, de Boer RA, DeMets D, Hernandez AF, et al. Dapagliflozin in Heart Failure with Mildly Reduced or Preserved Ejection Fraction. N Engl J Med. 2022;387(12):1089-98.

23. Pfeffer MA, Claggett B, Diaz R, Dickstein K, Gerstein HC, Køber LV, et al. Lixisenatide in Patients with Type 2 Diabetes and Acute Coronary Syndrome. N Engl J Med. 2015;373(23):2247-57.

24. Butler J, Jones WS, Udell JA, Anker SD, Petrie MC, Harrington J, et al. Empagliflozin after Acute Myocardial Infarction. N Engl J Med. 2024;390(16):1455-66.

25. Zinman B, Wanner C, Lachin JM, Fitchett D, Bluhmki E, Hantel S, et al. Empagliflozin, Cardiovascular Outcomes, and Mortality in Type 2 Diabetes. N Engl J Med. 2015;373(22):2117-28.

26. Anker SD, Butler J, Filippatos G, Ferreira JP, Bocchi E, Böhm M, et al. Empagliflozin in Heart Failure with a Preserved Ejection Fraction. N Engl J Med. 2021;385(16):1451-61.

27. Packer M, Anker SD, Butler J, Filippatos G, Pocock SJ, Carson P, et al. Cardiovascular and Renal Outcomes with Empagliflozin in Heart Failure. N Engl J Med. 2020;383(15):1413-24.

28. Zannad F, McMurray JJ, Krum H, van Veldhuisen DJ, Swedberg K, Shi H, et al. Eplerenone in patients with systolic heart failure and mild symptoms. N Engl J Med. 2011;364(1):11-21.

29. Holman RR, Bethel MA, Mentz RJ, Thompson VP, Lokhnygina Y, Buse JB, et al. Effects of Once-Weekly Exenatide on Cardiovascular Outcomes in Type 2 Diabetes. N Engl J Med. 2017;377(13):1228-39.

30. Sabatine MS, Giugliano RP, Keech AC, Honarpour N, Wiviott SD, Murphy SA, et al. Evolocumab and Clinical Outcomes in Patients with Cardiovascular Disease. N Engl J Med. 2017;376(18):1713-22.

31. Teerlink JR, Diaz R, Felker GM, McMurray JJV, Metra M, Solomon SD, et al. Cardiac Myosin Activation with Omecamtiv Mecarbil in Systolic Heart Failure. N Engl J Med. 2021;384(2):105-16.

32. Hernandez AF, Green JB, Janmohamed S, D'Agostino RB, Sr., Granger CB, Jones NP, et al. Albiglutide and cardiovascular outcomes in patients with type 2 diabetes and cardiovascular disease (Harmony Outcomes): a double-blind, randomised placebo-controlled trial. Lancet. 2018;392(10157):1519-29.

33. Cannon CP, Blazing MA, Giugliano RP, McCagg A, White JA, Theroux P, et al. Ezetimibe Added to Statin Therapy after Acute Coronary Syndromes. N Engl J Med. 2015;372(25):2387-97.

34. O'Donoghue ML, Glaser R, Cavender MA, Aylward PE, Bonaca MP, Budaj A, et al. Effect of Losmapimod on Cardiovascular Outcomes in Patients Hospitalized With Acute Myocardial Infarction: A Randomized Clinical Trial. Jama. 2016;315(15):1591-9.

35. Nidorf SM, Fiolet ATL, Mosterd A, Eikelboom JW, Schut A, Opstal TSJ, et al. Colchicine in Patients with Chronic Coronary Disease. N Engl J Med. 2020;383(19):1838-47.

36. Schwartz GG, Steg PG, Szarek M, Bhatt DL, Bittner VA, Diaz R, et al. Alirocumab and Cardiovascular Outcomes after Acute Coronary Syndrome. N Engl J Med. 2018;379(22):2097-107.

37. Investigators OT, Gerstein HC, Bosch J, Dagenais GR, Díaz R, Jung H, et al. Basal insulin and cardiovascular and other outcomes in dysglycemia. N Engl J Med. 2012;367(4):319-28.

38. Rao SV, Kirsch B, Bhatt DL, Budaj A, Coppolecchia R, Eikelboom J, et al. A Multicenter, Phase 2, Randomized, Placebo-Controlled, Double-Blind, Parallel-Group, Dose-Finding Trial of the Oral Factor XIa Inhibitor Asundexian to Prevent Adverse Cardiovascular Outcomes After Acute Myocardial Infarction. Circulation. 2022;146(16):1196-206.

39. Krahn AD, Longtin Y, Philippon F, Birnie DH, Manlucu J, Angaran P, et al. Prevention of Arrhythmia Device Infection Trial: The PADIT Trial. J Am Coll Cardiol. 2018;72(24):3098-109.

40. McMurray JJ, Packer M, Desai AS, Gong J, Lefkowitz MP, Rizkala AR, et al. Angiotensin-neprilysin inhibition versus enalapril in heart failure. N Engl J Med. 2014;371(11):993-1004.

41. Pfeffer MA, Claggett B, Lewis EF, Granger CB, Køber L, Maggioni AP, et al. Angiotensin Receptor-Neprilysin Inhibition in Acute Myocardial Infarction. N Engl J Med. 2021;385(20):1845-55.

42. Solomon SD, McMurray JJV, Anand IS, Ge J, Lam CSP, Maggioni AP, et al. Angiotensin-Neprilysin Inhibition in Heart Failure with Preserved Ejection Fraction. N Engl J Med. 2019;381(17):1609-20.

43. Bonaca MP, Bhatt DL, Cohen M, Steg PG, Storey RF, Jensen EC, et al. Long-term use of ticagrelor in patients with prior myocardial infarction. N Engl J Med. 2015;372(19):1791-800.

44. Wallentin L, Becker RC, Budaj A, Cannon CP, Emanuelsson H, Held C, et al. Ticagrelor versus clopidogrel in patients with acute coronary syndromes. N Engl J Med. 2009;361(11):1045-57.

45. Das Pradhan A, Glynn RJ, Fruchart JC, MacFadyen JG, Zaharris ES, Everett BM, et al. Triglyceride Lowering with Pemafibrate to Reduce Cardiovascular Risk. N Engl J Med. 2022;387(21):1923-34.

46. Van Gelder IC, Hagens VE, Bosker HA, Kingma JH, Kamp O, Kingma T, et al. A Comparison of Rate Control and Rhythm Control in Patients with Recurrent Persistent Atrial Fibrillation. New England Journal of Medicine. 2002;347(23):1834-40.

47. Swedberg K, Young JB, Anand IS, Cheng S, Desai AS, Diaz R, et al. Treatment of anemia with darbepoetin alfa in systolic heart failure. N Engl J Med. 2013;368(13):1210-9.

48. Bhatt DL, Steg PG, Miller M, Brinton EA, Jacobson TA, Ketchum SB, et al. Cardiovascular Risk Reduction with Icosapent Ethyl for Hypertriglyceridemia. N Engl J Med. 2019;380(1):11-22.

49. Metra M, Teerlink JR, Cotter G, Davison BA, Felker GM, Filippatos G, et al. Effects of Serelaxin in Patients with Acute Heart Failure. N Engl J Med. 2019;381(8):716-26.

50. Patel MR, Mahaffey KW, Garg J, Pan G, Singer DE, Hacke W, et al. Rivaroxaban versus warfarin in nonvalvular atrial fibrillation. N Engl J Med. 2011;365(10):883-91.

51. Scirica BM, Bhatt DL, Braunwald E, Steg PG, Davidson J, Hirshberg B, et al. Saxagliptin and cardiovascular outcomes in patients with type 2 diabetes mellitus. N Engl J Med. 2013;369(14):1317-26.

52. Swedberg K, Komajda M, Böhm M, Borer JS, Ford I, Dubost-Brama A, et al. Ivabradine and outcomes in chronic heart failure (SHIFT): a randomised placebo-controlled study. Lancet. 2010;376(9744):875-85.

53. Fox K, Ford I, Steg PG, Tardif JC, Tendera M, Ferrari R, Investigators S. Ivabradine in stable coronary artery disease without clinical heart failure. N Engl J Med. 2014;371(12):1091-9.

54. O'Donoghue ML, Braunwald E, White HD, Lukas MA, Tarka E, Steg PG, et al. Effect of darapladib on major coronary events after an acute coronary syndrome: the SOLID-TIMI 52 randomized clinical trial. Jama. 2014;312(10):1006-15.

55. Bhatt DL, Szarek M, Steg PG, Cannon CP, Leiter LA, McGuire DK, et al. Sotagliflozin in Patients with Diabetes and Recent Worsening Heart Failure. N Engl J Med. 2021;384(2):117-28.

56. Investigators S, White HD, Held C, Stewart R, Tarka E, Brown R, et al. Darapladib for preventing ischemic events in stable coronary heart disease. N Engl J Med. 2014;370(18):1702-11.

57. Nicholls SJ, Lincoff AM, Garcia M, Bash D, Ballantyne CM, Barter PJ, et al. Effect of High-Dose Omega-3 Fatty Acids vs Corn Oil on Major Adverse Cardiovascular Events in Patients at High Cardiovascular Risk: The STRENGTH Randomized Clinical Trial. Jama. 2020;324(22):2268-80.

58. Green JB, Bethel MA, Armstrong PW, Buse JB, Engel SS, Garg J, et al. Effect of Sitagliptin on Cardiovascular Outcomes in Type 2 Diabetes. N Engl J Med. 2015;373(3):232-42.

59. Steg PG, Bhatt DL, Simon T, Fox K, Mehta SR, Harrington RA, et al. Ticagrelor in Patients with Stable Coronary Disease and Diabetes. N Engl J Med. 2019;381(14):1309-20.

60. Morrow DA, Braunwald E, Bonaca MP, Ameriso SF, Dalby AJ, Fish MP, et al. Vorapaxar in the secondary prevention of atherothrombotic events. N Engl J Med. 2012;366(15):1404-13.

61. Tricoci P, Huang Z, Held C, Moliterno DJ, Armstrong PW, Van de Werf F, et al. Thrombin-receptor antagonist vorapaxar in acute coronary syndromes. N Engl J Med. 2012;366(1):20-33.

62. Roe MT, Armstrong PW, Fox KA, White HD, Prabhakaran D, Goodman SG, et al. Prasugrel versus clopidogrel for acute coronary syndromes without revascularization. N Engl J Med. 2012;367(14):1297-309.

63. Packer M, O'Connor C, McMurray JJV, Wittes J, Abraham WT, Anker SD, et al. Effect of Ularitide on Cardiovascular Mortality in Acute Heart Failure. N Engl J Med. 2017;376(20):1956-64.

64. Armstrong PW, Pieske B, Anstrom KJ, Ezekowitz J, Hernandez AF, Butler J, et al. Vericiguat in Patients with Heart Failure and Reduced Ejection Fraction. N Engl J Med. 2020;382(20):1883-93.

65. Nicholls SJ, Kastelein JJ, Schwartz GG, Bash D, Rosenson RS, Cavender MA, et al. Varespladib and cardiovascular events in patients with an acute coronary syndrome: the VISTA-16 randomized clinical trial. Jama. 2014;311(3):252-62.

66. Lincoff AM, Brown-Frandsen K, Colhoun HM, Deanfield J, Emerson SS, Esbjerg S, et al. Semaglutide and Cardiovascular Outcomes in Obesity without Diabetes. N Engl J Med. 2023;389(24):2221-32.
