# Supplementary material for: A comprehensive analysis of female participation in cardiovascular trials involving the WCN investigator network
Source: Neth Heart J. 2025 Nov 12;33(12):404–11. doi: 10.1007/s12471-025-01999-4 (PMC12638513; doi:10.1007/s12471-025-01999-4)
Supplement: Supplementary file 5 — Supplemental Fig. 1. Flowchart of the completed cardiovascular trials [file 12471_2025_1999_MOESM5_ESM.docx]

Trials excluded:

- No information on ClinicalTrials.gov and no availability of an original article (n = 35)
- Open-label extension study (n = 7)
- Observational study (n = 6)
- Primary prevention trials (n = 2)

Completed WCN trials (n = 165)

Trials excluded for analysis:

- No stratification by sex for the absolute number of events (n = 20)

Included trials for analysis of the percentage of women reaching study endpoint (n = 46)

Included trials for analysis of the statistical weight and logREM (n = 66)

Trials excluded for analysis:

- No event-driven studies (n = 29)
- No stratification by sex for the REM (n = 18)
- Primary safety endpoint (n = 2)

Included trials for analysis of the PPR (n = 115)
