# Supplementary figures and images for: A comprehensive analysis of female participation in cardiovascular trials involving the WCN investigator network
Source: Neth Heart J. 2025 Nov 12;33(12):404–11. doi: 10.1007/s12471-025-01999-4 (PMC12638513; doi:10.1007/s12471-025-01999-4)

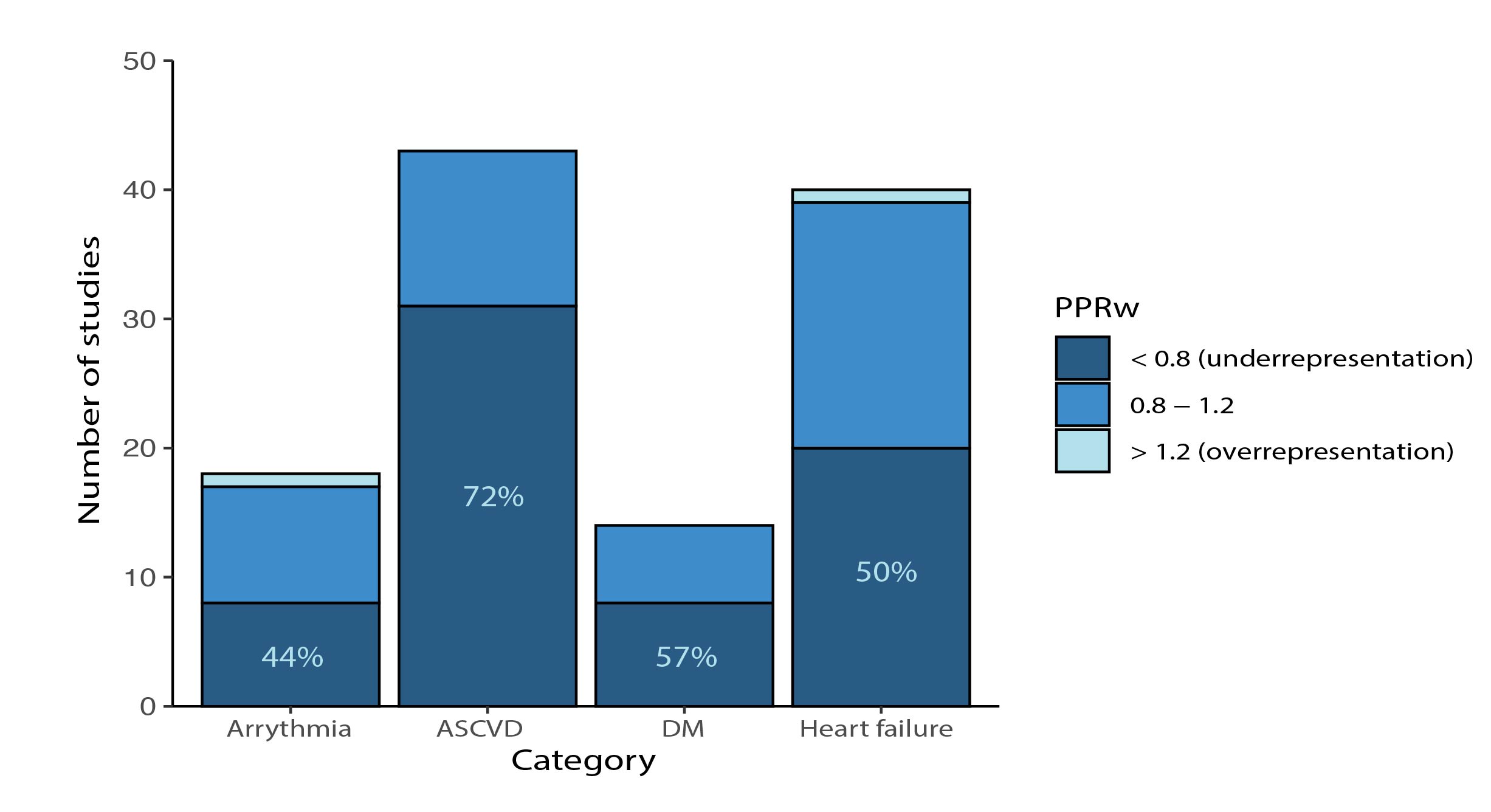

Supplement: Supplementary file 6 — Supplemental Fig. 2. Participation to Prevalence Ratios (PPR) per trial category [file 12471_2025_1999_MOESM6_ESM.docx]

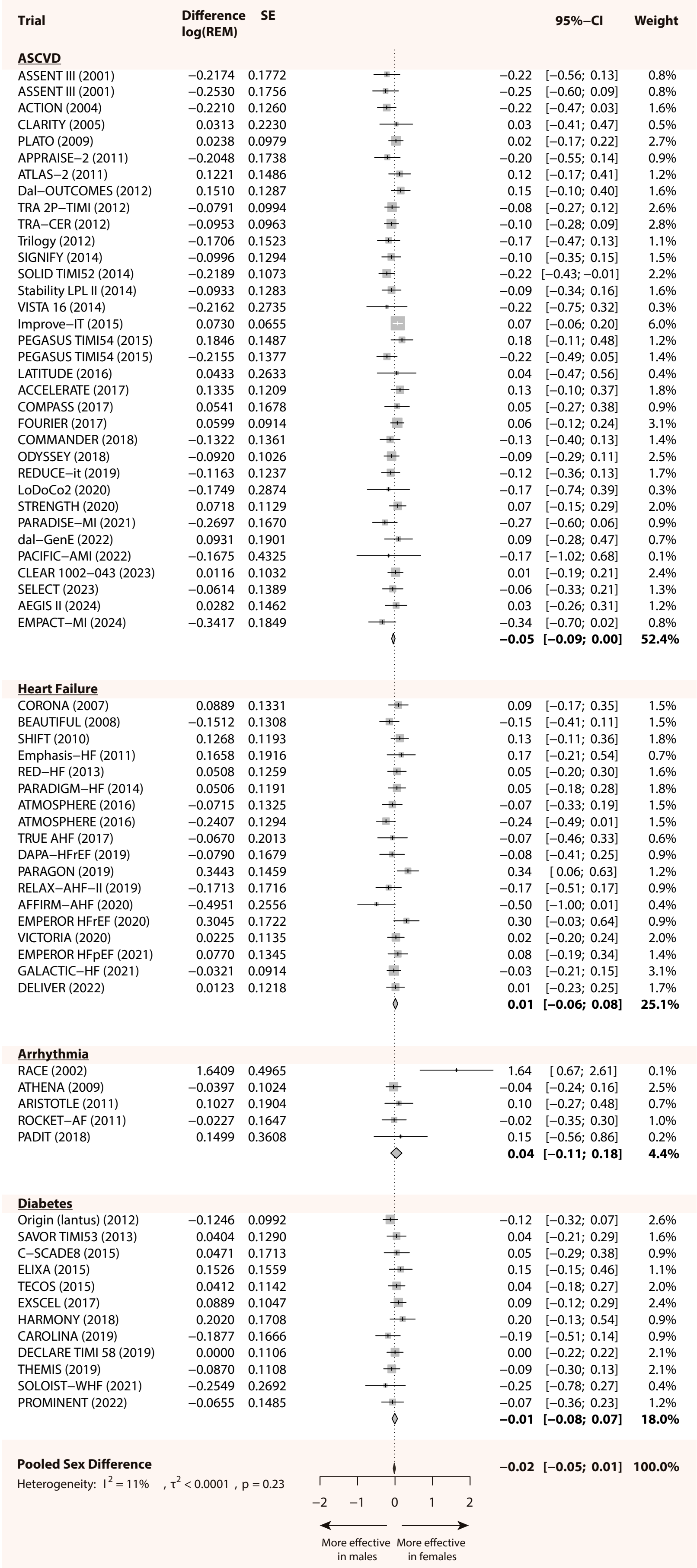

Supplement: Supplementary file 7 — Supplemental Fig. 3. Forest plot difference log(REM) [file 12471_2025_1999_MOESM7_ESM.docx]

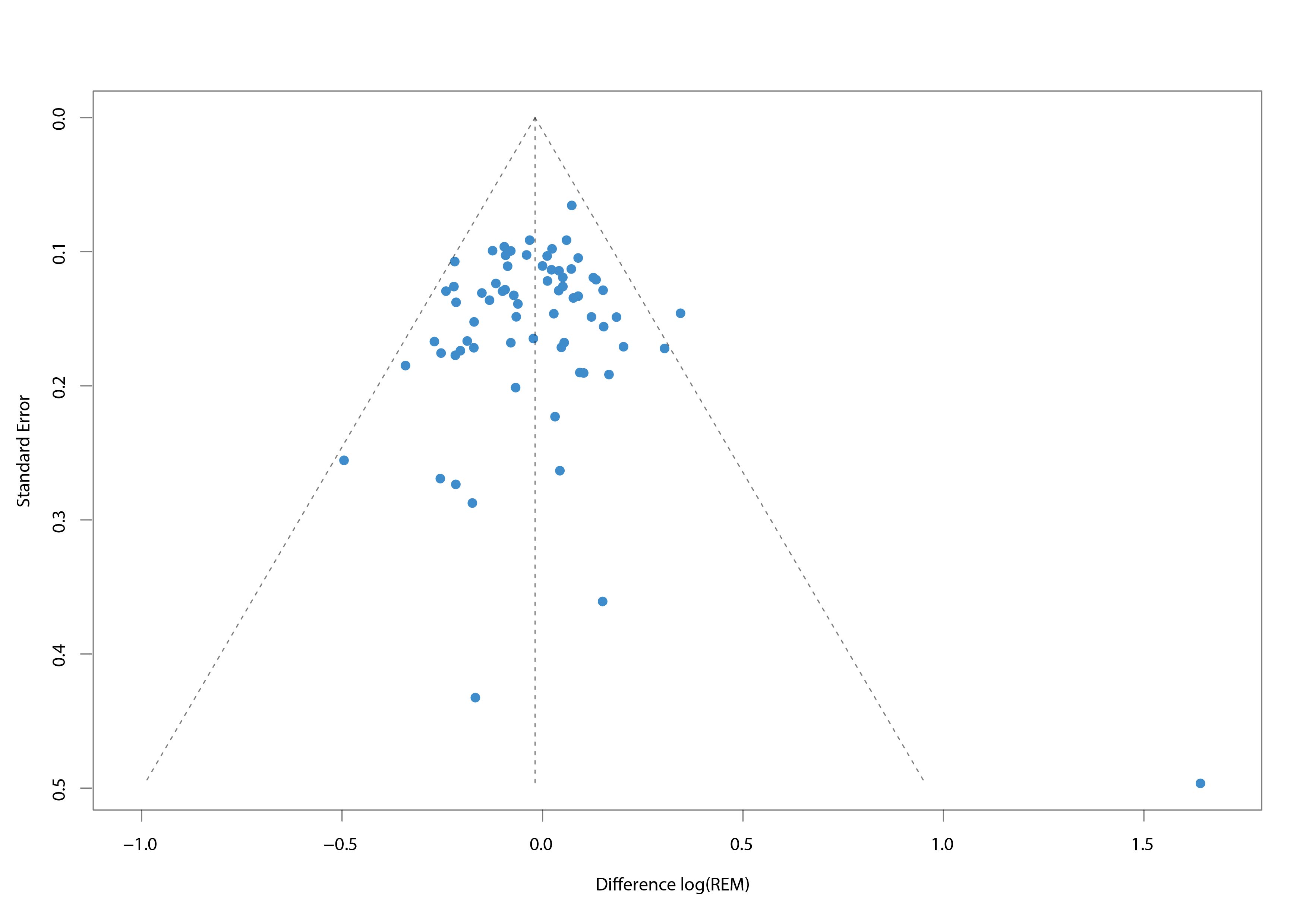

Supplement: Supplementary file 8 — Supplemental Fig. 4. Funnel plot difference log(REM) [file 12471_2025_1999_MOESM8_ESM.docx]
